# Small-molecule properties define partitioning into biomolecular condensates

**DOI:** 10.1038/s41557-024-01630-w

**Published:** 2024-09-13

**Authors:** Sabareesan Ambadi Thody, Hanna D. Clements, Hamid Baniasadi, Andrew S. Lyon, Matthew S. Sigman, Michael K. Rosen

**Affiliations:** 1 Department of Biophysics, Howard Hughes Medical Institute, UT Southwestern Medical Center, Dallas, TX USA; 2 Department of Chemistry, University of Utah, Salt Lake City, UT USA; 3 Department of Biochemistry, UT Southwestern Medical Center, Dallas, TX USA

**Keywords:** Biophysical chemistry, Mass spectrometry

## Abstract

Biomolecular condensates regulate cellular function by compartmentalizing molecules without a surrounding membrane. Condensate function arises from the specific exclusion or enrichment of molecules. Thus, understanding condensate composition is critical to characterizing condensate function. Whereas principles defining macromolecular composition have been described, understanding of small-molecule composition remains limited. Here we quantified the partitioning of ~1,700 biologically relevant small molecules into condensates composed of different macromolecules. Partitioning varied nearly a million-fold across compounds but was correlated among condensates, indicating that disparate condensates are physically similar. For one system, the enriched compounds did not generally bind macromolecules with high affinity under conditions where condensates do not form, suggesting that partitioning is not governed by site-specific interactions. Correspondingly, a machine learning model accurately predicts partitioning using only computed physicochemical features of the compounds, chiefly those related to solubility and hydrophobicity. These results suggest that a hydrophobic environment emerges upon condensate formation, driving the enrichment and exclusion of small molecules.

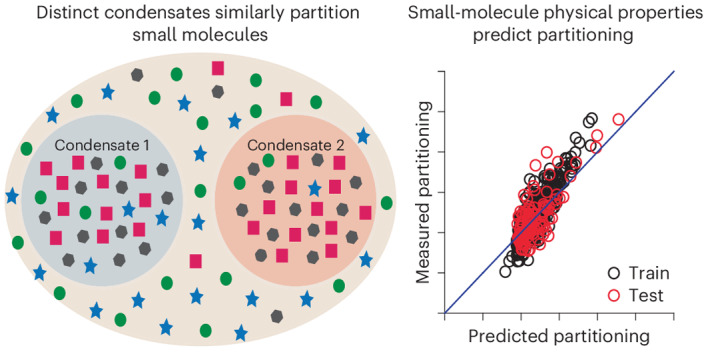

## Abstract (web summary)

Biomolecular condensates compartmentalize molecules without membranes. Understanding condensate composition is important given that their function relies on the selective exclusion or enrichment of molecules. Now, investigating small-molecule partitioning reveals variations across compounds, yet correlations indicate physical similarities between disparate condensates. Machine learning accurately predicts partitioning on the basis of physicochemical features, demonstrating the role of a hydrophobic environment in driving enrichment and exclusion.

## Main

Biomolecular condensates are cellular compartments that concentrate proteins, nucleic acids and likely small molecules in the absence of an encapsulating membrane^1,2^. Many condensates appear to form through liquid–liquid phase separation of multivalent macromolecules^1–3^. Condensates are involved in numerous biological processes, including signal transduction, intermediary metabolism, RNA metabolism and gene regulation. Aberrant condensates are associated with human diseases including neurodegeneration, cancer and viral infections^4–6^, and bioinformatic analyses have recently implicated condensate dysregulation in over 1,000 genetic disorders^7^. Condensates have thus emerged as therapeutic targets^6,8,9^. Drug development efforts seek to identify small molecules that disrupt condensates or modulate their material properties, or that alter the activity of specific macromolecular components.

Condensates are believed to segregate, concentrate and modulate biochemical processes in vivo, affording specificity and efficiency to individual reactions and pathways^5,10,11^. Understanding the mechanisms that determine condensate composition is thus important for defining condensate function. In models of macromolecular composition, many proteins and RNAs are recruited through specific binding to other condensate components^12–15^. In addition, proteins can be enriched in or excluded from condensates without apparent site-specific binding, in a manner that depends on a protein’s overall charge^16–18^. Less is understood about the factors that govern the composition of small organic molecules in condensates, although recent efforts have begun to characterize such factors^19–21^. Multiple mechanisms are likely to be important and can be conceptualized on a spectrum of binding affinities and specificities. At one end, high-affinity, stereospecifically defined interactions can occur between a compound and a complementary element in a macromolecular condensate component. These will drive recruitment of the compound into the condensate, as has been observed for many macromolecules. By contrast, low-affinity, low-specificity interactions with solvent and/or other molecules can govern the distribution of a compound between the distinct biochemical environments inside and outside a condensate. Such factors can lead to the enrichment or exclusion of a compound from the condensate phase. It remains unknown which types of interaction play dominant roles in dictating the recruitment of small molecules into biomolecular condensates.

Here we examined the partitioning of a large collection of small-molecule metabolites and pharmaceuticals into four different condensates composed of unrelated proteins and protein–DNA mixtures, both purified and in cell extracts. The degree of partitioning spans nearly six orders of magnitude, from ~100-fold exclusion to ~10,000-fold enrichment. Surprisingly, partitioning is correlated among the different condensates, indicating a similarity in their underlying physical properties. Several highly partitioning compounds do not show measurable interactions with one model protein system under non-phase-separating conditions, suggesting that enrichment is not due to specific binding to condensate components. We developed a machine learning model, whose only inputs are physicochemical properties of the compounds (and not the molecular structures), that predicts values of the partition coefficient (PC) with quantitative accuracy in both the pure protein and extract systems, providing potential routes to drugs that target condensates. Furthermore, the model indicates that the strongest predictors of partitioning behaviour are aqueous solubility and other measures related to hydrophobicity. Whereas high-affinity binding to condensate macromolecules will also contribute to enrichment, and may account for some inaccuracies of the model, such effects are not dominant for the systems under study. The experimental data and modelling suggest that the chemical properties of small molecules largely define their partitioning behaviours, such that compounds with enhanced hydrophobic character favour concentration into the condensates. This enrichment of organic compounds in the condensate is an emergent biochemical property of the phase-separated compartment, which is not found in the individual macromolecules.

## Results

### Diverse partitioning with correlations between condensates

We examined the partitioning of small molecules into four disparate condensates composed of proteins and nucleic acids. We characterized two synthetic condensates, one composed of polySH3 and polyproline-rich motif (PRM) proteins^22^ (that is, SH3PRM) and the other composed of polySUMO and polySUMO-interaction motif (SIM) proteins^12^ (that is, SUMOSIM). In addition, we examined two condensates formed by naturally occurring macromolecules, one formed by the yeast P-body protein Dhh1 (ref. ^13^) (that is, Dhh1) and the other formed by the innate immune signalling protein cyclic GMP-AMP synthase (cGAS) and double-stranded DNA^23^ (that is, cGASDNA).

**Fig. 1:**
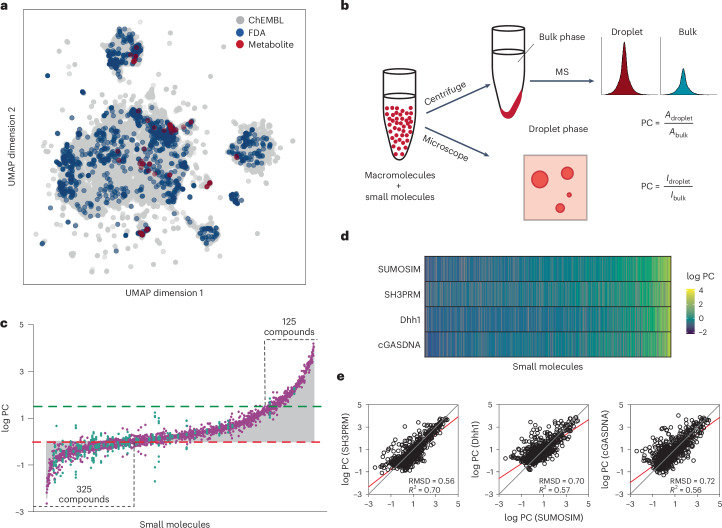
Partitioning of chemical compounds varies over nearly six orders of magnitude and is correlated between condensates. **a**, UMAP representation of ~1,700 small molecules used in the analysis that is based on physical features generated in QikProp. **b**, Schematic illustrating the assays used to measure small-molecule partitioning into condensates on the basis of mass spectrometry (MS; top) and confocal fluorescence microscopy (bottom). In **b**, *A*_droplet_ and *A*_bulk_ are the area under the curve for droplet and bulk fractions measured using MS, respectively. Similarly, *I*_droplet_ and *I*_bulk_ are the fluorescence intensity from droplets and bulk solution measured using confocal fluorescence microscopy, respectively. **c**, Bar chart of PC values, ordered from smallest to largest, for the partitioning of 1,037 compounds into the SUMOSIM condensate. Red and green dashed lines indicate log PC = 0 and log PC_SUMOSIM_ (1.77), respectively. The numbers of compounds with log PC < 0 and log PC > log PC_SUMOSIM_ are indicated. In **c**, the grey-coloured areas represent the bar plots for the mean values of the data, and green and purple dots represent metabolites and drug compounds, respectively. **d**, Heat map showing the log PC values for each of the four condensates indicated. Columns are organized in ascending order according to the average PC value across the four condensates. **e**, Scatter plots of log PC values for compounds into the SUMOSIM condensate versus into the SH3PRM, Dhh1 and cGASDNA condensates. Red and grey lines show linear fit of the data and diagonal, respectively. Source data

**Extended Data Fig. 1:**
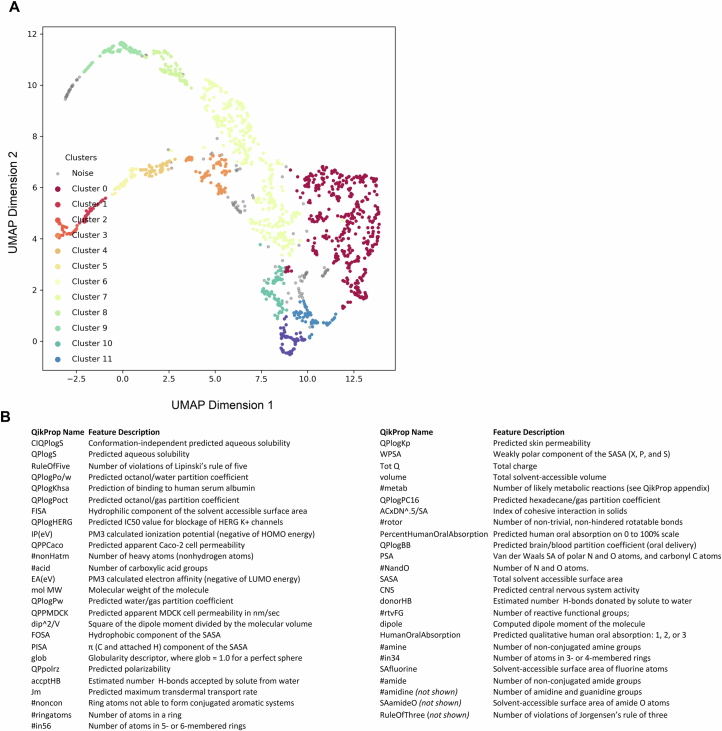
Chemical space map of all the compounds used in this study. **a**. Color-coded static chemical space map of all the compounds used in this study. UMAP dimensions 1 and 2 are abstract coordinates that combine QikProp features to visualize the relationships among the compounds. An interactive chemical space map of all compounds used in this study can be accessed using Supplementary Data 1 (Interactive HTML file). Hovering over data points shows the corresponding name and structure; molecules are colored according to their cluster. **b**. Definitions of QikProp feature names used throughout text. Source data

For each condensate, we assessed the partitioning of two libraries of molecules, one of ~200 metabolite compounds and one with ~1,500 drug compounds approved by the US Food and Drug Administration (FDA). To assess the diversity of the compounds, we combined them with 200,000 randomly chosen biologically active small molecules from the ChEMBL database, and calculated ~50 ADME (absorption, distribution, metabolic and excretion) and chemical properties for each using the QikProp software package^24^. The multidimensional descriptor set was embedded into a two-dimensional chemical space using the Uniform Manifold Approximation and Projection (UMAP) algorithm^25^. As illustrated in Fig. 1a, the compounds used in our study distribute relatively evenly across the chemical space representation mapped by the ChEMBL compounds, illustrating the diversity of our library. When UMAP was applied solely to our library, clustering analysis using an unsupervised learning algorithm (HDBSCAN)^26^ identified 11 groups of small molecules with similar chemical attributes, suggesting that QikProp descriptors convey enough chemical information to discriminate between classes of molecules (Extended Data Fig. 1—an interactive map can be accessed in Supplementary Data 1). There are well-defined isolated clusters for steroidal compounds, guanidine-containing compounds and β-lactam antibiotics. The other clusters share boundaries but have distinct characteristics. The largest of these clusters contains small biomolecules, multi-chlorinated aryl compounds and common non-steroidal anti-inflammatory drugs with a variety of structures. Overall, the UMAP and clustering indicate a diverse but relatively sparsely populated set of molecules, with a few classes (for example, steroids and β-lactams) that are more densely sampled.

**Extended Data Fig. 2:**
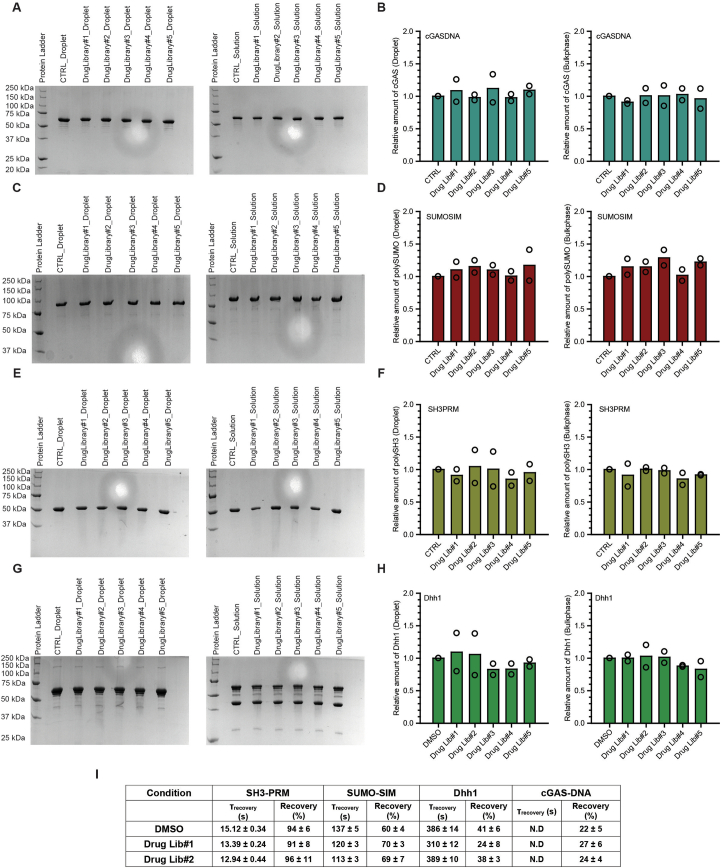
Compound libraries do not appreciably affect scaffold protein partitioning or condensate dynamics. **A**-**I**. Quantification of the amount of scaffold protein in the condensate and bulk phases in the absence and presence of different compound libraries using an SDS-PAGE method. The figures (**A**) for cGAS-DNA, (**C**) for SUMOSIM, (**E**) for SH3-PRM, and (**G**) for Dhh1 show example raw gel images of proteins in condensate and bulk fractions in the presence of DMSO control or one of five ~300-compound sublibraries of FDA approved drugs. The corresponding relative (to control) quantification of scaffold protein in the condensate fractions is displayed in (B) for cGAS-DNA, (**D**) for SUMOSIM, (**F**) for SH3-PRM, and (**H**) for Dhh1. Data distribution shown for two independent experiments. The data show that within experimental error the amounts of scaffold in both the condensate and bulk phases did not change with addition of drugs. It remains formally possible that droplet volumes and partition coefficients are changing in concert (inversely, such that total material is unchanged), but it is unlikely that this would be the case in all four condensates with all 5 chemical sub libraries. We also note anecdotally we did not observe obvious differences in droplet size or morphology between the DMSO controls and drug library experiments. Thus, the simplest interpretation for the data is that the compounds do not affect the condensate volume or partition coefficients of the scaffolds. **I**. Fluorescence Recovery After Photobleaching (FRAP) of all four condensates in the absence (DMSO) and presence of two drug sublibraries (Dark-Library #1 and Dark-Library #2, derived from standard sublibraries #1 and #2 by removing fluorescent compounds). The data provided in Supplementary Fig. 1. The time of recovery and the percentage of recovery for each condensate system under different conditions are presented in the table below the figure. Errors are the standard deviation calculated from at least 3 independent measurements. Source data

We developed two assays to measure the partitioning of the compound libraries (Fig. 1b). In the first, we incubated sublibraries of ~300 compounds, each at 1 µM concentration, with the macromolecules under phase-separating conditions (2–10 µM concentrations). As described in Extended Data Fig. 2, the compound libraries did not substantially alter the volume, PC or dynamics of the condensates (Supplementary Fig. 1). After equilibration (1–24 h), the condensates were collected via centrifugation followed by manual separation of the pellet and supernatant. Compounds were subsequently extracted from the two samples and quantified using mass spectrometry, yielding the PC as the ratio of concentrations in the pellet and supernatant. Analysis of replicate measurements revealed that the average percentage error in the measured PC values was less than 20% for all of the condensates (SUMOSIM = 19.5%, SH3PRM = 13.6%, Dhh1 = 10.8%, cGASDNA = 10.0%), and that the percentage error is consistent across the range of PC values.

**Extended Data Fig. 3:**
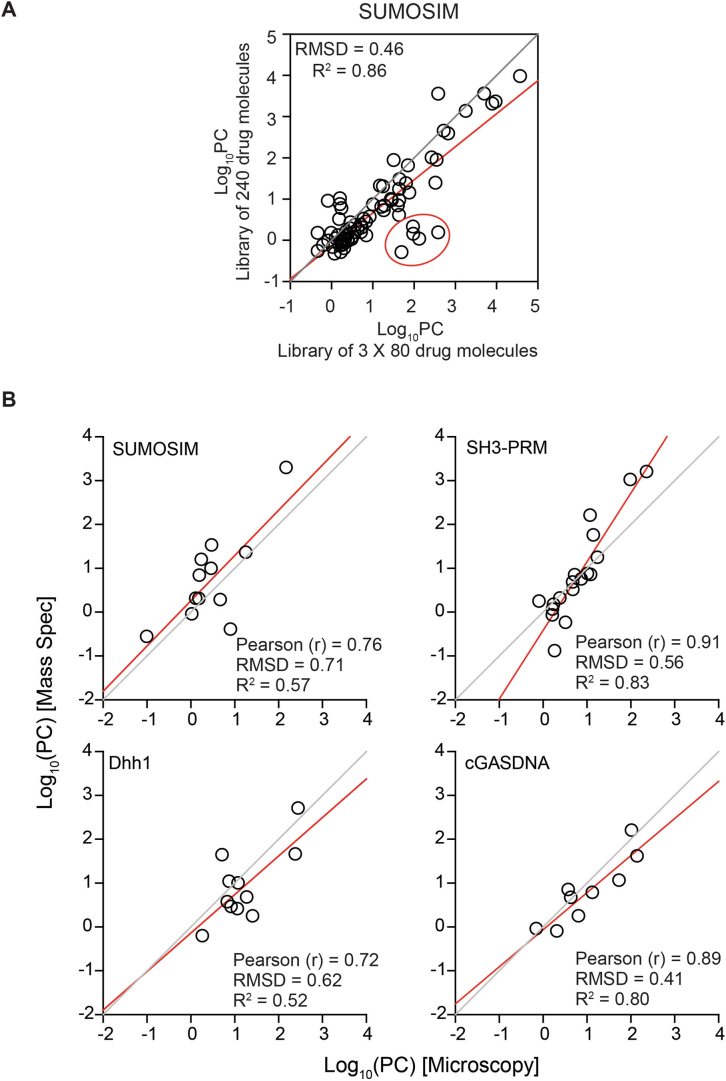
Most compounds have similar PC values when measured in large or smaller groups. (**a**) Scatter plot of logPC values measured for a library of 240 compounds versus the values for the same compounds divided into three groups of 80 species. Other than the five compounds circled, most molecules have very similar logPC values in the larger or smaller groups (R2 = 0.86, excluding the circled outliers). (**b**) The mass spectrometry and confocal fluorescence microscopy assays of partition coefficients yield similar values for most species. Plots show correlations between logPC values measured by mass spectrometry and confocal fluorescence microscopy for the indicated condensates. Red and grey lines show linear fit of the data and diagonal, respectively. Source data

Our pooled-library approach relies on each compound behaving independently, with the compounds not substantially modulating the exclusion or enrichment of others. To examine this, we analysed the partitioning into SUMOSIM condensates of a sublibrary that contained 240 drug molecules and the same molecules divided into three subgroups of 80 compounds each. Apart from a few outliers (five molecules), the PCs measured in each case were similar (coefficient of determination (*R*^2^) = 0.86; Extended Data Fig. 3a). Thus, whereas we cannot entirely rule out cross-talk between certain molecules, the compounds generally behave independently from each other in this assay.

To validate the mass spectrometry results, we used confocal fluorescence microscopy with the 30 fluorescent molecules in the drug library individually incubated with macromolecules under phase-separating conditions. PCs were determined from the ratio of fluorescence intensity values within the condensate and the surrounding solution. In each condensate, PCs measured using mass spectrometry and microscopy were reasonably correlated (*R*^2^ = 0.52–0.83, root mean squared deviation (RMSD) = 0.41–0.71; Extended Data Fig. 3b). The modest number of outliers was probably due to changes in the fluorescence intensity of certain compounds in the dense and dilute phases^27^ or to cross-talk between the pooled molecules in the mass spectrometry assay.

Figure 1c plots the PC values of the metabolite and drug libraries in the SUMOSIM condensate measured via the mass spectrometry assay. The values span a nearly 1,000,000-fold range, from ~0.01 to 10,000, with the strongest partitioning corresponding to a free energy of ~5.6 kcal mol^−1^ for transfer of a compound into the condensate from the dilute phase. Of the 1,037 compounds that could be reliably measured (Supplementary Methods), 247 had PC values of <0.8, indicating that they are substantially excluded from the condensates; 224 compounds had PC values in the range of 0.8–1.5, indicating little preference for condensates versus surroundings; and 566 had PC values of 1.5–10,000, indicating modest to strong enrichment in the condensates. Given the droplet volume fraction of 1.2 ± 0.2% and the 1 µM total compound concentration, the highest enrichment corresponds to a concentration of ~90 µM in the condensate. The metabolites populate the lower end of the PC range, spanning 0.14–35, whereas the drugs sample the entire range of values.

**Extended Data Fig. 4:**
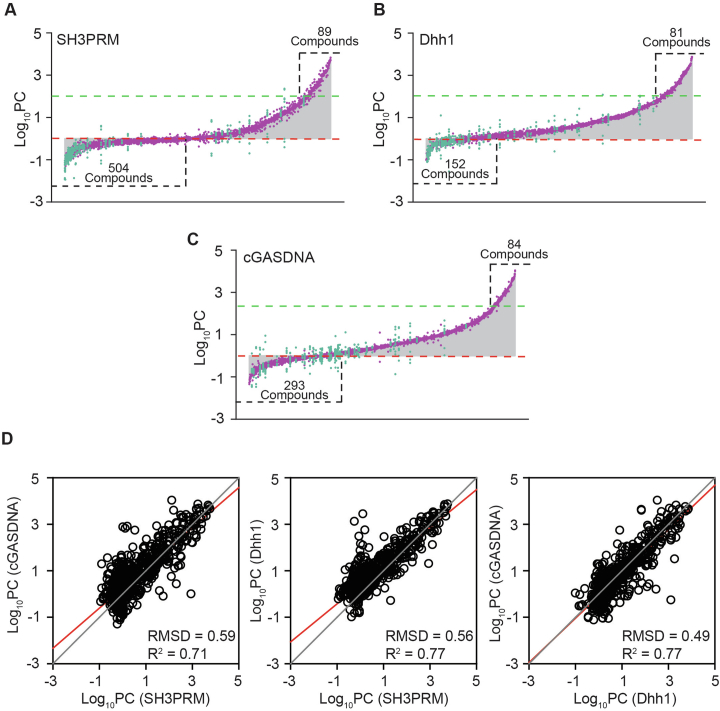
Each condensate shows a wide range of partition coefficients for the small molecules. Scatter plot of partition coefficients, ordered from smallest to largest, for 1296, 1213, and 1296 compounds into (**a**) SH3PRM, (**b**) Dhh1 and (**c**) cGASDNA condensates, respectively. In each panel the red dotted lines indicate logPC = 0 and green dotted lines indicate logPCscaffold (2.0, 2.1, and 2.3, respectively). The grey-colored areas represent bar plots for the mean values of the data, and green and purple dots represent metabolites and drug compounds, respectively. (**d**) Partitioning of small molecules is correlated between the different condensates. Scatter plots of logPC values for compounds into cGASDNA versus SH3PRM condensates, Dhh1 versus SH3PRM condensates, and Dhh1 versus cGASDNA condensates. Red and grey lines show linear fit of the data and diagonal, respectively. Source data

**Extended Data Fig. 5:**
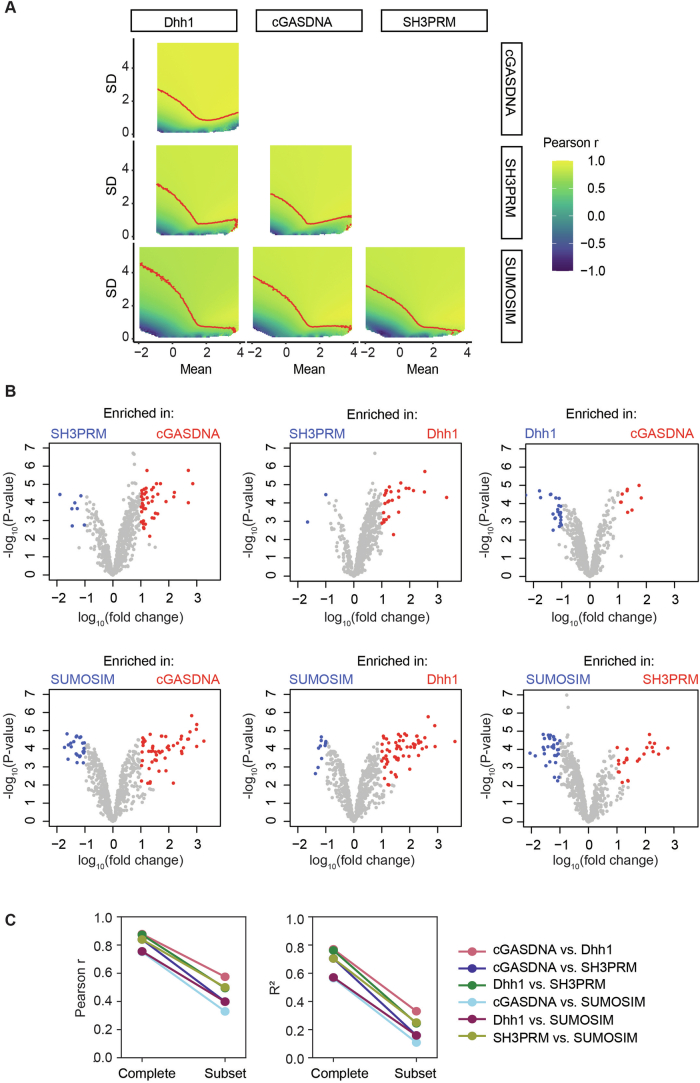
Correlations in subsampled windows between PCs of small molecules in different condensate systems. In panel **A**, each pixel in the raster images represents the Pearson correlation coefficient r between PC values of small molecules in each pair of condensate systems, with the PC data subsampled with a normally distributed bias with mean and SD parameters as indicated on the x- and y-axes, respectively. The red contour line indicates the r value for the complete (that is, not subsampled) dataset. The images are thus a qualitative representation of the range of PC values over which correlations between each pair of condensate systems become apparent. The color scale is the same in all images. (**B**) Differential partitioning of compounds in each pair of condensate systems. Each plot shows -log10(p-value) as a function of log10(fold-change) in PC for each compound that was detected in all four condensate systems (977 compounds). Compounds were considered significantly differentially enriched in a given condensate if P < 0.01 and fold change in PC > 10 and are colored red or blue. Compounds that did not exhibit significant differential partitioning are colored gray. (**C**) Pearson r and R2 values for each condensate PC comparison values calculated for complete PC range and subset of PC values between 0.9 and 13. Source data

**Extended Data Fig. 6:**
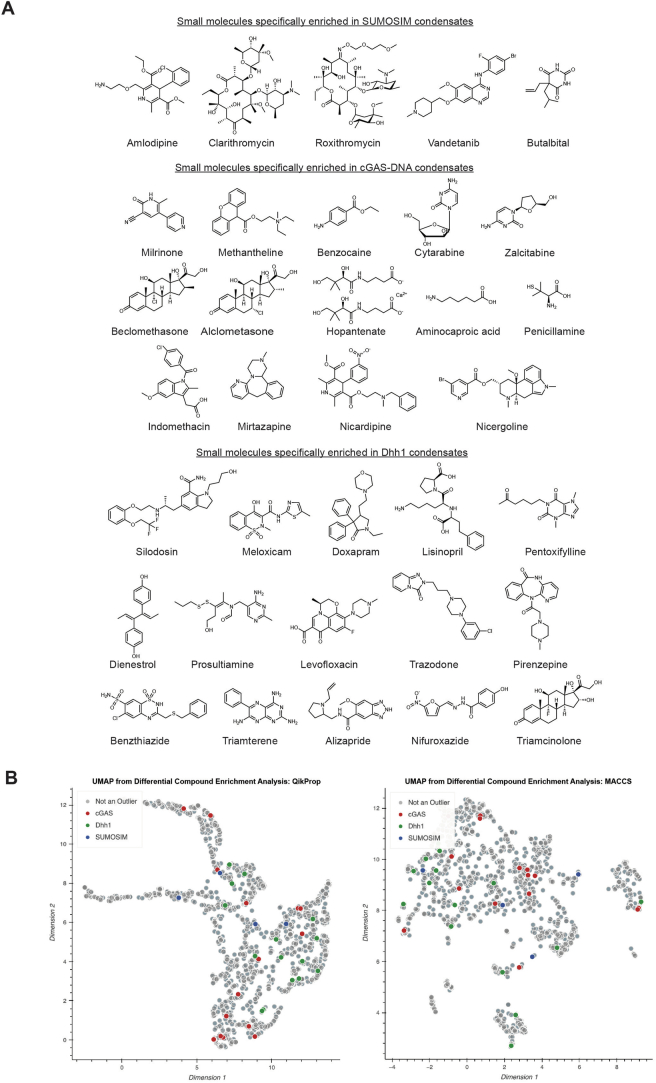
Specifically enriched compounds. (**a**) Based on the data in Extended Data Fig. 5, the figure illustrates the compounds that are specifically enriched in the SUMOSIM, cGASDNA and Dhh1 condensates relative to all other condensates (see Supplementary Methods for analysis methods). No compounds were specifically enriched in the SH3PRM condensates. (**b**) A UMAP based on QuikProp features showing small molecules that showed specific enrichment in various condensates. Out of the library of metabolites and FDA-approved drugs, sixteen compounds showed specific enrichment in cGAS condensates (Red), 15 in Dhh1 condensates (green) and five in SUMOSIM condensates (blue). There were no small molecules that were specifically enriched in SH3PRM condensates (Supplementary Table 12). Compounds without specific enrichment in any condensate are colored grey. The small molecules occupy distinct regions within chemical space, suggesting the absence of common physicochemical attributes responsible for differential enrichment among specific condensate systems. Note the UMAP here differs from that in Extended Data Fig. 1 because it was generated with a different initial seed. UMAP analysis of specifically enriched compounds based on MACCS keys. Source data

We initially anticipated that the individual condensate systems would show different distributions of partitioning as they are composed of unrelated molecules with different physical properties (Supplementary Tables 1 and 7). Surprisingly, however, the different condensates showed similar ranges of PC values for the small molecules (Fig. 1d and Extended Data Fig. 4), and the distributions are strongly correlated between the different condensates over the full range of PC values (Fig. 1e and Extended Data Fig. 4). Correlations are weaker when considering smaller ranges of PC values (less than ~1–2 log units; Extended Data Fig. 5), and may indicate that effects specific to each macromolecule govern minor differences in PC (see the Discussion). We also analysed outliers in these correlations to identify molecules that showed specificity for an individual condensate over the three others (Extended Data Figs. 5 and 6). Within each specificity group, members did not share common physical or structural features (Extended Data Fig. 6). Together, these results indicate that, despite the differences in macromolecular components, there is an underlying physical similarity between the condensates such that the same small molecules tend to be enriched or excluded by each condensate system.

### Stereospecific binding alone does not drive partitioning

**Extended Data Fig. 7:**
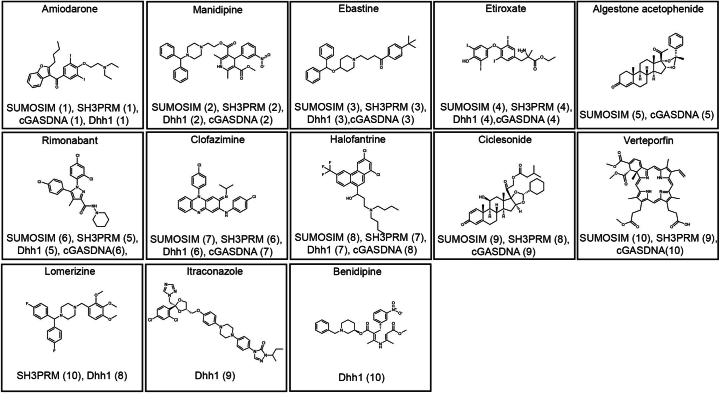
Chemical structures of the ten most strongly partitioning compounds for each of the four condensates. The collection of strongly partitioning molecules exhibits a diverse range of functional groups, lacking a singular shared structural motif across all compounds. Among the small molecules, the inclusion of unsaturated or aromatic rings is prevalent, although certain condensates display considerable enrichment of linear, highly saturated molecules (for example, Alexidine in SH3PRM condensates). Both straightforward ring systems, exemplified by Ethoxyquin, and intricate, stereochemically complex ring systems like Algestone acetophenide (and other steroids) demonstrate a pronounced capacity for substantial enrichment. Notably, polarizable functional groups such as nitro groups and halogens emerge as favored moieties within the highly enriched molecules. Nonetheless, their presence is not required for strong enrichment. These findings indicate that the general basis of partitioning is not governed by stereospecific interactions between small molecules and condensates.

Several features of our PC dataset suggest that small-molecule partitioning is not governed by specific binding to the condensate macromolecular components. First, for enrichment due to stereospecific binding to a discrete site, PC_compound_ is bounded by 1 and PC_scaffold_, assuming that the macromolecular scaffolds have equal affinity for a compound in the dense and dilute phases^12^. Yet here, each condensate has numerous compounds that are either substantially excluded (PC < 1) or have PC values that far exceed those of the macromolecular components (Fig. 1c and Extended Data Fig. 4a). Second, whereas PC_compound_ values can lie outside these limits if the macromolecular components have different affinities for compounds inside and outside the condensate^28^, the similarity of distributions between the different condensates argues against this possibility (Fig. 1e and Extended Data Fig. 4b); it is unlikely that two independent scaffolds would both possess stereospecific binding sites for a compound that are differentially populated to similar degrees in the dense and dilute phases. Finally, the highly partitioning compounds for each condensate, as well as the specifically enriched compounds for each condensate, do not possess common structural features, as would be expected if they recognized a specific binding site in the scaffold (Extended Data Figs. 6 and 7). Thus, the data suggest that partitioning is not driven by stereospecific binding to condensate scaffolds but rather by the general physical properties of the compounds, which differentially favour the dense or dilute phases.

**Fig. 2:**
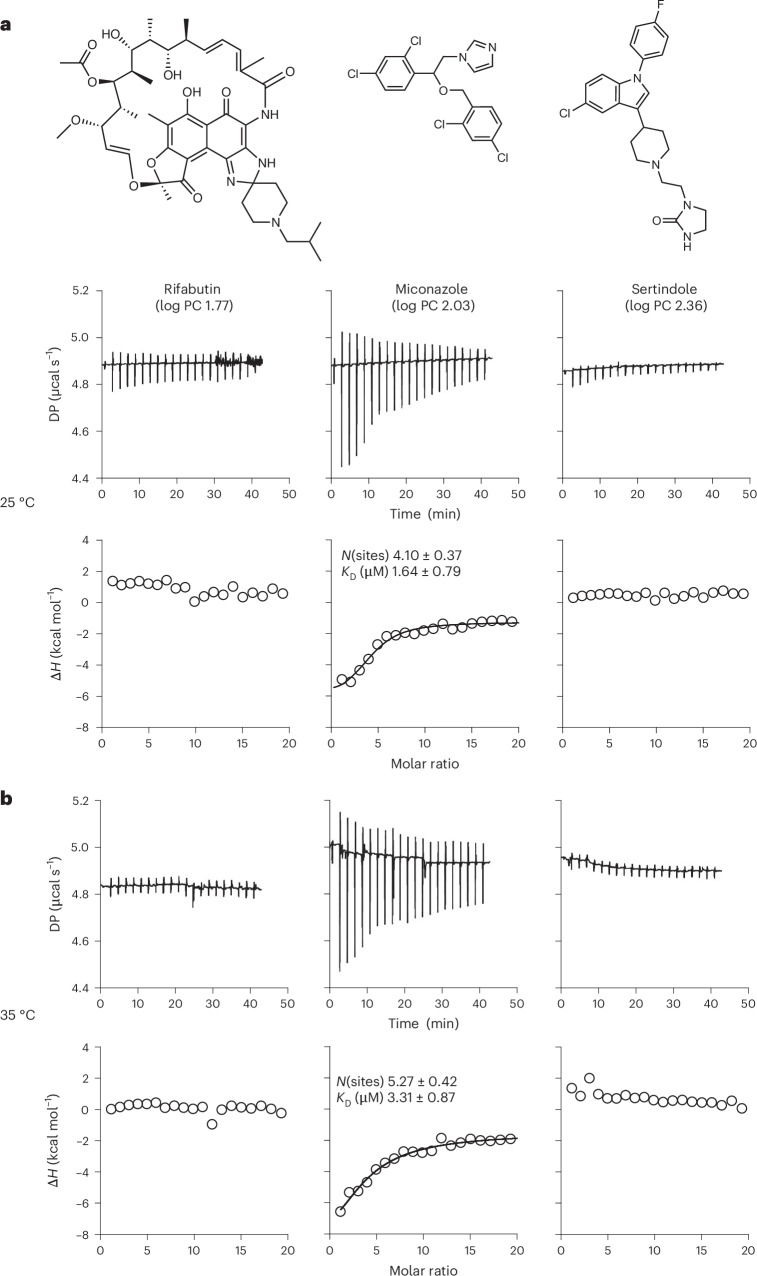
Many compounds do not bind SUMOSIM scaffolds under non-phase-separating conditions. **a**,**b**, Raw ITC thermograms (top row) and integrated enthalpies (bottom row) for titrations of rifabutin (left), miconazole (middle) and sertindole (right) into 20 µM module concentrations of polySUMO + polySIM (below the phase separation threshold) at 25 °C (**a**) and 35 °C (**b**). DP, differential power. Source data

To test this hypothesis, we used isothermal titration calorimetry (ITC) to examine the binding of 13 compounds to the polySUMO–polySIM complex at a concentration below the phase separation threshold (20 µM module concentration). These compounds had PC values ranging from 50 to 4,000 and were titrated up to 200 µM, a tenfold excess over the scaffolds. As illustrated in Fig. 2 and Supplementary Fig. 2, at both 25 and 35 °C, nine of the compounds showed essentially flat titration profiles, with an enthalpy of binding (Δ*H*) near zero. The absence of measurable heat at two different temperatures strongly suggests the absence of binding rather than a small heat of binding. Three of the compounds showed relatively flat profiles with non-zero enthalpies, suggesting a weak interaction that is far from saturation even at a tenfold excess of ligand. Only miconazole showed saturable binding, with a dissociation constant (*K*_D_) value in the low micromolar range. Thus, most of the compounds examined do not have a measurable affinity for the polySUMO–polySIM complex in the absence of phase separation. Even for miconazole, its PC value is well above that of the scaffolds (107 versus 60), suggesting that binding alone cannot account for its enrichment into the SUMOSIM condensate. These experiments further support the hypothesis that, for most compounds, partitioning is driven primarily by the physical properties of the compounds and condensates rather than stereospecific binding to scaffolds.

### Predicting compound partitioning from physical properties

We turned to statistical modelling to determine which chemical features of small molecules determine partitioning, with a related goal of exploiting the models to predict the partitioning behaviour of new compounds. We used the chemical space map and clustering to define balanced training and test sets for modelling and validation purposes. We withheld 20% of the molecules from each cluster for external validation and used an additional 20% as a test set to compare machine learning algorithms. The remaining 60% of the molecules were selected as the training set to develop models. For SUMOSIM condensates, confidence intervals were computed via tenfold re-randomization of the train–test split. Model exploration included the evaluation of different data structures and machine learning algorithms through comparison of the test set *R*^2^ and mean absolute error (MAE) in the predicted log PC of the resultant models (see Supplementary Methods for modelling details).

**Fig. 3:**
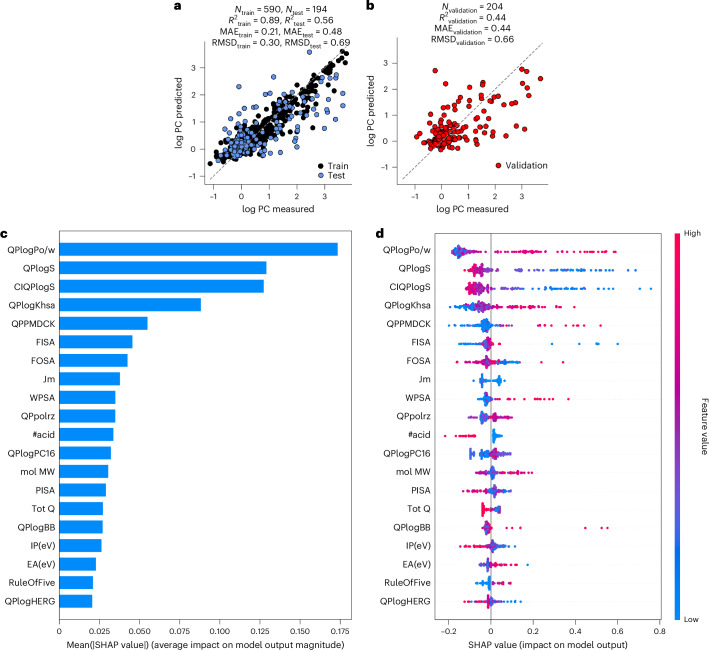
Machine learning models for the partitioning of small molecules into biomolecular condensates are predictive and indicate that physical features determine the partitioning behaviour. A 100-fold re-randomization of train–test sets was used to compute the indicated confidence intervals. **a**, XGBoost model of small-molecule partitioning into SUMOSIM condensates (*N*_train_ = 590 (black), *N*_test_ = 194 (blue), *R*^2^_train_ = 0.89 ± 0.01, MAE_train_ = 0.21 ± 0.01, *R*^2^_test_ = 0.56 ± 0.03, MAE_test_ = 0.48 ± 0.01). **b**, Validation set predictions for the SUMOSIM model (*N*_train_ = 784 (not shown), *N*_validation_ = 204 (red), *R*^2^_validation_ = 0.44 ± 0.05, MAE_validation_ = 0.44 ± 0.03). **c**, SHAP feature importance analysis showing the relative feature importance of the top SUMOSIM XGBoost model. QPlogPo/w, predicted octanol/water partition coefficient; QPlogS, predicted aqueous solubility; CIQPlogS, conformation-independent predicted aqueous solubility; QPlogKhsa, prediction of binding to human serum albumin; QPPMDCK, predicted apparent MDCK cell permeability; FISA, hydrophilic component of the solvent-accessible surface area; FOSA, hydrophobic component of the solvent-accessible surface area; Jm, predicted maximum transdermal transport rate; WPSA, weakly polar component of the solvent-accessible surface area; QPpolrz, predicted polarizability in cubic ångströms; #acid, number of carboxylic acid groups; QPlogPC16, predicted hexadecane/gas partition coefficient; mol MW, molecular weight of the molecule; PISA, *π* (carbon and attached hydrogen) component of the solvent-accessible surface area; Tot Q, total charge; QPlogBB, predicted brain/blood partition coefficient; IP(eV), PM3-calculated ionization potential; EA(eV), PM3-calculated electron affinity; RuleOfFive, number of violations of Lipinski’s rule of five; QPlogHERG, predicted IC_50_ value for blockage of HERG K^+^ channels. **d**, SHAP decomposition showing that the impact of features on each model prediction shows nonlinearity between small-molecule feature values and the measured partitioning behaviour. Source data

**Extended Data Fig. 8:**
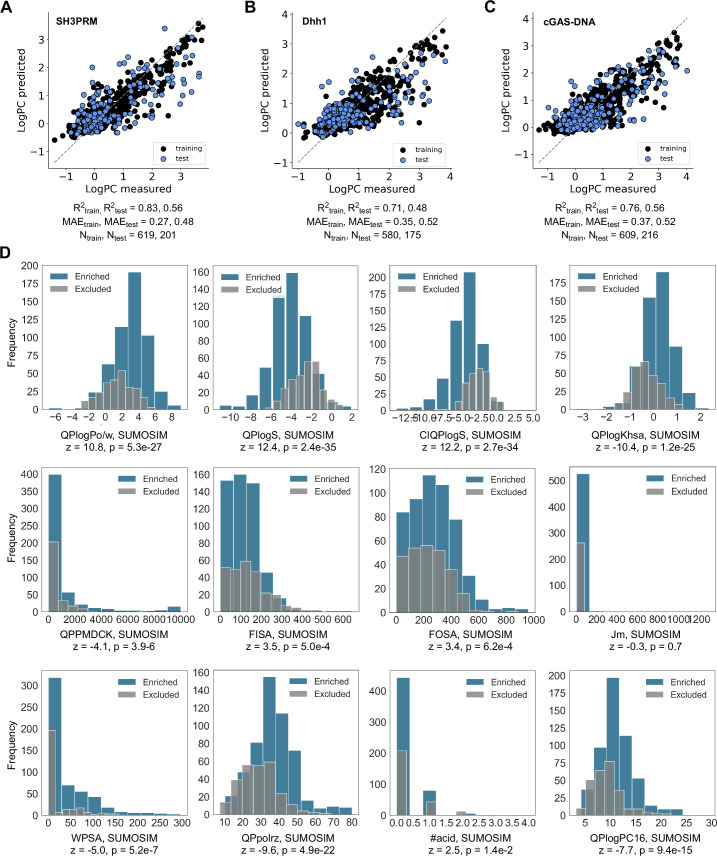
Extreme gradient boosting (XGBoost) models of other condensates. Shown are models of (**a**) SH3PRM, (**b**) Dhh1, and (**c**) cGASDNA, where the black line shows linear fit of the diagonal. MAE = mean absolute error. (**d**) Z-test comparison of enriched (N = 651) vs. excluded (N = 307) molecules for the top features in SUMOSIM model. Enriched molecules (blue) had measured log10PC greater than zero and excluded molecules (gray) had measured log10PC less than or equal to zero. Source data

Extreme gradient parallel tree boosting (XGBoost) models generally outperformed other algorithms^29^, and QikProp descriptors outperformed structure-based molecular encodings that produce a larger feature set (Extended Data Fig. 1 and Supplementary Information). Combining QikProp with structure-based encodings did not improve the models, whereas adding descriptors of the condensate scaffolds (Supplementary Table 8) led to overfitting; these features were not used in the final models. The XGBoost model of SUMOSIM partitioning had a modest *R*^2^_test_ value of 0.56 and a log PC MAE_test_ of 0.48 (Fig. 3a and Supplementary Table 9). Models for the other condensate systems performed similarly, with *R*^2^_test_ in the range of 0.48–0.58 and log PC MAE_test_ in the range of 0.44–0.52 (Extended Data Fig. 8 and Supplementary Table 9).

Given the strong correlation of PC values across the condensate systems, we reasoned that the performance of our models should not depend strongly on which condensate PC dataset is used for model training. To address this, we compared the individual models with one based on the combined data for all four condensates using a single condensate descriptor in addition to the QikProp features (Supplementary Table 9). As hypothesized, this combined model performed similarly to the models of each condensate, with *R*^2^_test_ and MAE_test_ values of 0.56 and 0.50, respectively. We also constructed a model based on the average log PC for each molecule across all condensates, a dataset of comparable size to the individual condensate models (Supplementary Table 10, entry 6). The average log PC model had an overall *R*^2^_test_ of 0.58 and a MAE_test_ of 0.44, a notable improvement compared with the individual condensate and combined models. Together, these findings suggest that condensate–small-molecule interactions are mostly conserved with respect to the condensate identity. This emergent quality implies that, among the condensates examined here, the condensate composition plays a secondary role in small-molecule partitioning.

We validated the average log PC model by predicting the withheld data. The model successfully predicted the partitioning behaviour for the validation set within one-half of a log PC unit (*R*^2^_validation_ = 0.44, MAE_validation_ = 0.44; Fig. 3b), corresponding to a PC error of less than threefold. Although the validation predictions plateau near log PC = 0 (probably due to the limited experimental range of log PC for excluded molecules compared with enriched molecules), the MAE of the excluded validation points is only 0.33 log PC units. We recognize that the sparsity of data relative to the molecular diversity surveyed resulted in the observed undertraining, and we aim to supplement our database of small-molecule partitioning measurements to explore this limitation in future studies. Prediction errors did not substantially change as a function of which cluster a compound originated from (Extended Data Fig. 1), suggesting that the model effectively covers the diverse compound representation. Furthermore, only 12% of molecules had prediction errors greater than one log PC unit, showing that our model successfully differentiates highly enriched molecules from those that are weakly attracted to or excluded from the condensates. We anticipate that the ability to predict differences in partitioning across nearly six orders of magnitude will have substantial benefit for future applications.

**Extended Data Fig. 9:**
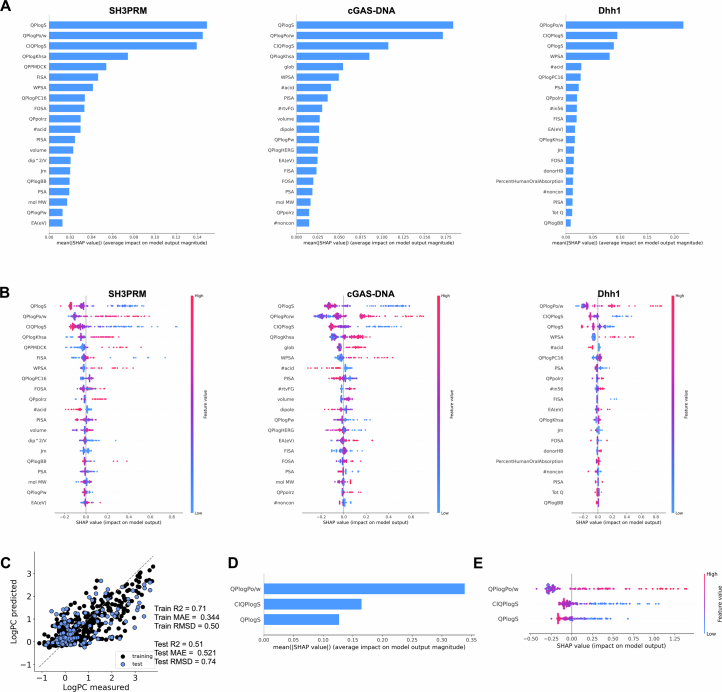
Shapley Additive Explanations (SHAP) feature importance analysis of the three additional condensates. (**a**) Feature importance is computed as the average impact on the model predictions when the specified feature is neglected from the XGBoost model. (**b**) SHAP decomposes each prediction into the contributions of each feature, which reveals the relative importance of different features and their interactions in determining specific outcomes from the XGBoost models. (**c**) XGBoost model trained on top features of SUMOSIM buffer model. (**d**) The six most important QikProp descriptors (as indicated by SHAP values, Fig. 3) resulted in an XGB model that had slightly poorer statistics than the analogous model trained on all QikProp features (Test R2 = 0.44 vs 0.47, test MAE = 0.51 vs 0.49). (**e**) Comparison of SHAP analysis of the two models indicated that the SHAP value magnitudes were similar across models, although there were minor fluctuations in the ordering in feature importance. Source data

**Extended Data Fig. 10:**
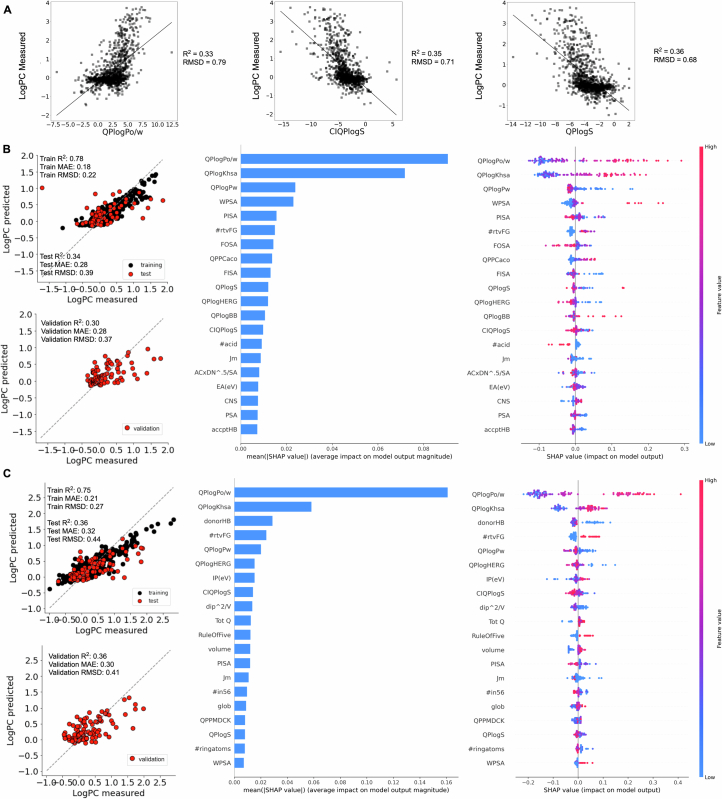
Univariate linear regression models of small molecules partitioning. (**a**) The top features identified from the full XGBoost model of the SUMOSIM condensate were subjected to univariate linear regression analysis. The resultant models demonstrated that linear fits to top descriptors are not sufficient to capture the impact of small molecules partitioning into SUMOSIM condensates in buffer. (**b**) Analysis of small molecule data for SUMOSIM condensates in U2OS cell lysate. XGBoost model of small molecules partitioning into SUMOSIM condensates in U2OS cell lysate (N train = 320 (black), N test = 131 (red), train R2 = 0.78+/− 0.01, train MAE = 0.18+/− 0.01, test R2 = 0.34+/− 0.11, test MAE = 0.28+/− 0.03). Validation set predictions (N train = 451 (not shown), N validation = 90 (red), validation R2 = 0.42+/− 0.07, validation MAE = 0.4451, +/− 0.03). Shapley Additive Explanations (SHAP) feature importance analysis showing the relative feature importance of the top SUMOSIM U2OS lysate model. SHAP decomposition showing the impact of features on each model prediction shows nonlinearity between small molecule feature values and measured partitioning behavior. (**c**) Analysis of small molecule data for SUMOSIM condensates in Xenopus laevis oocyte extract. XGBoost model of small molecules partitioning (N train = 366 (black), N test = 162 (red), train R2 = 0.75+/− 0.01, train MAE = 0.21+/− 0.02, test R2 = 0.36+/− 0.06, test MAE = 0.32+/− 0.02). B. Validation set predictions (N train = 528 (not shown), N validation = 106 (red), validation R2 = 0.36+/− 0.09, validation MAE = 0.30, +/− 0.02). Shapley Additive Explanations (SHAP) feature importance analysis showing the relative feature importance of the top SUMOSIM Xenopus laevis oocyte extract model. SHAP decomposition showing the impact of features on each model prediction shows nonlinearity between small molecule feature values and measured partitioning behavior. Source data

*Z*-test comparisons of the top features in the SUMOSIM model demonstrated a significant difference in the distributions of enriched versus excluded small molecules (Extended Data Fig. 8). Building on these differences, we used SHAP (SHapley Additive exPlanations) analysis^30^ to decompose each prediction into the contributions of each feature, to reveal the relative importance of the different features and their interactions in determining specific outcomes from the XGBoost model (Fig. 3c,d and Extended Data Fig. 9a,b). Both the two-sample Z-test and SHAP analyses revealed that, for all condensates, features describing the aqueous solubility (CIQPlogS and QPlogS) and oil/water partitioning (QPlogPo/w) were the most influential in predicting small-molecule partitioning, along with the capacity to engage promiscuous, hydrophobic binding sites in proteins (QPlogKhsa)^31,32^. These features are all generally associated with hydrophobicity. Conservation of feature importance across models of the four individual condensates suggests that the guidelines of small-molecule partitioning are relatively consistent for all condensates (Extended Data Fig. 9). As described in Extended Data Fig. 9, we also generated an XGBoost model for the SUMOSIM condensate using only the three most important QikProp descriptors, and found that this model had slightly poorer statistics than the model trained on all QikProp features (*R*^2^_test_ = 0.51 versus 0.56, MAE_test_ = 0.52 versus 0.48). None of the top features alone is strongly correlated with the PC, indicating the importance of the collection (Extended Data Fig. 10a). Thus, solubility and hydrophobicity effects dominate the predictive models of partitioning in a nonlinear fashion, while additional physicochemical descriptors can offer some fine-tuning of the predictions.

In addition to the QikProp features, we explored the possibility that structural features, such as molecular fingerprints, may outperform physiochemical representations of small molecules. Therefore, we computed two types of molecular fingerprint^33–35^ for the SUMOSIM dataset. As demonstrated in Supplementary Table 10 and discussed in the Supplementary Methods, QikProp features outperformed both robust (ECFP4 fingerprints) and compact (MACCS keys) representations of small molecules. Thus, physical properties, rather than specific chemical structures, are most predictive of partitioning behaviour (in the absence of stereospecific binding—see the Discussion).

Together, these observations provide further support for the hypothesis that partitioning in this compound library is largely driven by the physical properties of the molecules, as opposed to stereospecific binding.

### Solution conditions can influence compound partitioning

**Fig. 4:**
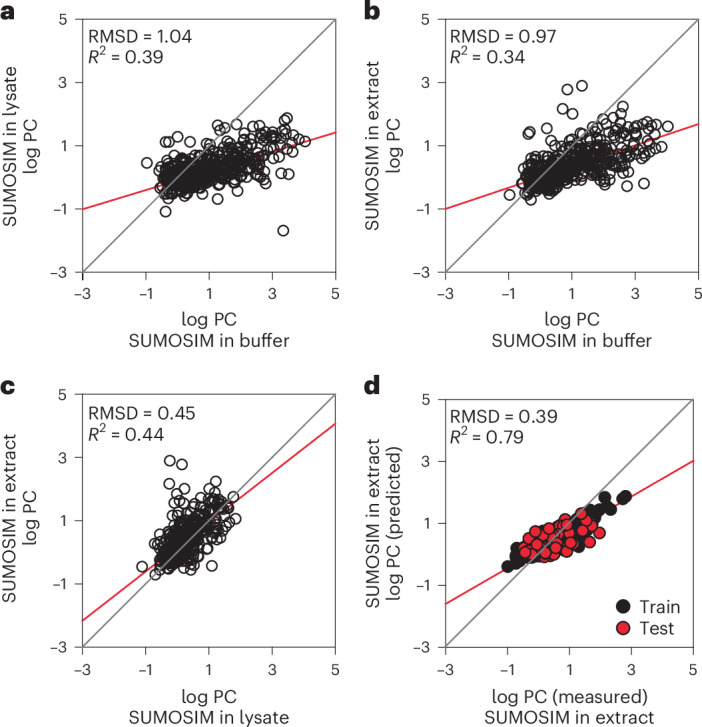
Compounds show different distributions of PC values under different solvent conditions. **a**,**b**, Scatter plots of log PC values of compounds in SUMOSIM condensates generated in a U2OS cell lysate (**a**) or a *Xenopus* oocyte extract (**b**) versus in buffer. **c**, Scatter plot of log PC values of compounds in SUMOSIM condensates generated in the *Xenopus* oocyte extract versus in the U2OS cell lysate. **d**, Machine learning model of compound partitioning in the *Xenopus* oocyte extract. Red and grey lines show linear fit of the data and diagonal, respectively. Source data

Our results suggest that the chemical environment within a condensate is different from the surrounding solution, and this difference plays an important role in determining whether organic compounds are enriched or excluded. However, the vastly more complex environment of the cell relative to simple buffers may impact partitioning in ways that are not accounted for by our experiments thus far. To address this, we quantified partitioning of the drug library into SUMOSIM condensates under conditions that are designed to mimic the cellular environment, either in a relatively dilute U2OS cell lysate (~4 mg ml^−1^ total protein) or a highly concentrated *Xenopus laevis* oocyte extract (80–100 mg ml^−1^, comparable to the eukaryotic cytoplasm). As shown in Fig. 4a,b, for both systems, the partitioning behaviours remain modestly correlated to those observed for SUMOSIM condensates in simple buffer (with *R*^2^ values of 0.39 and 0.34, respectively). However, the total range of PC values decreases by ~100-fold, spanning ~0.1–1,000, with only a few values larger than 100. The reduced range is probably due in part to a roughly tenfold decrease in PC values for the polySUMO and polySIM proteins themselves (60 in buffer, 8 in U2OS lysate, 5 in *Xenopus* extract). In addition, the much greater complexity of the lysate/extract solution probably dampens the differences between dense and dilute phases. On average, the log PC values decrease around twofold between the buffer and either lysate, as indicated by the slopes of the lines correlating the distributions and the relatively large RMSD in the log PC values between the datasets (1.04 and 0.97, respectively, for buffer versus lysate and buffer versus extract). Although the lysate and extract have disparate total protein concentrations, their effects on compound partitioning are similar, with an *R*^2^ of 0.44 and an RMSD of 0.45 log PC units between the datasets (Fig. 4c). Thus, the differences between the buffer and lysate/extract environments appear to stem more from the number of molecular species rather than the concentrations of those species.

As with the pure condensate systems, we were able to generate a features-based statistical model of compound partitioning into the SUMOSIM condensates in a *Xenopus* extract system (Fig. 4d). The model was somewhat less predictive in the more complex solution than in the simple buffer (*R*^2^_validation_ = 0.39, MAE_validation_ = 0.30), although this deficit may be due to the reduced number of compounds that can be analysed due to technical limitations (Supplementary Information and Supplementary Table 6) rather than an inherent limitation of modelling extract systems. The features driving the model in the extract system shared similarities with the buffer model: octanol/water partitioning and features related to hydrophobicity are still dominant, although measures of aqueous solubility are not important in the extract model (Extended Data Fig. 10b,c). Together, the data and modelling demonstrate that, even in highly complex solution environments that mimic the cytoplasm, the drugs show a wide range of PC values, which can be predicted by their physical properties.

## Discussion

We have demonstrated that the partitioning of small molecules into condensates varies over nearly six orders of magnitude, from modest exclusion to strong enrichment. The combination of biochemical data and statistical modelling indicates that, in most cases, partitioning is largely driven by the aqueous solubility and hydrophobic character of compounds rather than their ability to stereospecifically bind to discrete sites on condensate scaffolds. In general, though, for compounds that do bind scaffolds through specific chemical structures, both effects should contribute to partitioning and could reinforce or oppose each other depending on their relative signs; the net behaviour will depend on both their signs and magnitudes. An unexpected similarity of partitioning profiles among several different condensates suggests that phase separation results in a shared, general physical or chemical property among the macromolecules that we have characterized. This property differentiates the physical environment within condensates from that of the surrounding solution, leading to enrichment or exclusion. The physical basis of the difference in environment remains unclear but may involve the solvent structure or ionic composition within the condensates or perhaps a greater abundance of transient, partially unfolded states of proteins that could create hydrophobic surfaces for recruiting small molecules non-stereospecifically^36,37^.

Kilgore et al.^19,21^ recently described a ‘chemical grammar’ model for small-molecule partitioning, wherein distinct chemical environments occur in different condensates, which enhance the partitioning of molecules bearing specific chemical moieties. By contrast, our observations suggest a model of overall chemical similarity between condensates, with partitioning driven by compatibility between the physical properties of compounds and the similar chemical environments of the condensates. Key differences between the studies lie in the compound libraries and corresponding PC ranges. Kilgore et al.^21^ use a library based on three intrinsically fluorescent molecules that were diversified via combinatorial synthesis, which afforded a 15-fold variation in PC (0.9–13). By contrast, we used a library of metabolites and drugs that were not constructed around a common molecular core, which produced around a million-fold variation (~0.01–10,000). For data in the PC range of 0.9–13, we also observe substantial differences between condensates, with Pearson correlation coefficients ranging from 0.33 to 0.57, rather than 0.75–0.87 observed with our complete dataset (Extended Data Fig. 5c; see also Extended Data Figs. 6 and 7). Thus, within narrow PC windows, condensates show distinct patterns of partitioning, probably due to subtle variations in chemical environment and/or weak binding of scaffolds to compounds. But when sampling a wide range of chemical space and PC values, overall correlations in partitioning and thus global similarities in chemical environment between condensates are revealed. Our model suggests that physical properties are better predictors of partitioning than specific chemical structures. A limitation of our data is that all condensates we examined are formed by proteins with folded domains, whereas intrinsically disordered proteins and nucleic acids can also form condensates. Thus, it remains possible that other types of condensate have more distinct chemical environments. Future experiments on diverse condensate systems will help to clarify the generality and limitations of our observation for similar chemical environments among the four condensate systems studied here.

A variety of approaches have been suggested for modulating the activities and/or physical properties of condensates to impact human diseases^6,8,10^. The machine learning methods that we deployed here could accelerate these approaches by facilitating the rapid identification of highly enriching compounds. In diseases where therapeutic targets reside in condensates, modelling could guide the rational modification of existing inhibitors to increase their concentrations in the compartments. Existing drugs could also be grafted onto strongly partitioning compounds to direct them into condensates; both of these strategies could provide increased potency or selectivity towards targets. It is important to note that enrichment via the site-specific binding of a small molecule to one condensate component does not imply a thermodynamic advantage for binding any other component. Thus, the potency of a drug that targets one condensate component may not improve simply by adding moieties that specifically bind another component. However, our finding that strong enrichment can be achieved without stereospecific binding, potentially due to solvent properties, suggests that drug potency could be enhanced by an overall increase in compound concentration within the condensate not localized at any specific binding sites.

The physical properties of condensates that form through phase separation, such as viscoelasticity and surface tension, are emergent. That is, they are inherently macroscopic, resulting from interactions of the parts of the system but not manifest in the parts individually^38^. Here we have found that the ability to recruit small molecules appears to be an emergent biochemical property of SUMOSIM condensates. Twelve of the 13 drugs analysed using ITC do not bind the polySUMO–polySIM complex tightly in the absence of phase separation but are recruited strongly into SUMOSIM condensates. Thus, for these compounds the free energy of partitioning emerges only upon phase separation. Given that the compounds analysed via ITC were selected randomly (Supplementary Methods), this emergent behaviour is probably operative for many compounds. It may also occur for other condensates. Moreover, we have previously demonstrated that the membrane dwell time, and consequently the signalling activity, is higher in Nephrin/Nck/N-WASP condensates than in small complexes of the same molecules, again emerging as a function of size^39^. Future work on how the biochemical activity that is resident in condensates varies as a function of the condensate size will reveal the extent to which emergent biochemistry is a general feature of condensates in biology.

### Reporting summary

Further information on research design is available in the Nature Portfolio Reporting Summary linked to this article.

## Online content

Any methods, additional references, Nature Portfolio reporting summaries, source data, extended data, supplementary information, acknowledgements, peer review information; details of author contributions and competing interests; and statements of data and code availability are available at 10.1038/s41557-024-01630-w.

## Supplementary information

Supplementary Information Supplementary Methods, Figs. 1–3, Table 1 and Methods-only references.

Reporting Summary

Supplementary Table 2 List of metabolites used in this study.

Supplementary Table 3 List of drug molecules used in this study.

Supplementary Table 4 List of fluorescent drugs and fluorophores used in this study.

Supplementary Table 5 Raw area-under-the-curve values for all four condensate systems.

Supplementary Table 6 Data details of data filtering.

Supplementary Table 7 PC values of all the condensates used in this study.

Supplementary Table 8 Amino acid parameters of all four condensate proteins.

Supplementary Table 9 Comparison of statistics of all algorithms used in this study.

Supplementary Table 10 Details of XGBoost model statistics.

Supplementary Table 11 Details of XGBoost model parameters.

Supplementary Table 12 Details of complete differential enrichment analysis.

Supplementary Table 13 Details of compound correlation statistics.

Supplementary Software 1 R code used for differential enrichment data analysis.

Supplementary Data 1 Interactive UMAP supplementary to Extended Data Fig. 1a.

Supplementary Data 2 Excel files used for each plot.

Supplementary Data 3 Excel files used for each plot.

Supplementary Data 4 Excel files used for each plot.

## Source data

Source Data Fig. 1 Excel files used for each plot.

Source Data Fig. 2 Excel files used for each plot.

Source Data Fig. 3 Excel files used for each plot.

Source Data Fig. 4 Excel files used for each plot.

Source Data Extended Data Fig. 1 Additional HTML files for interactive UMAP.

Source Data Extended Data Fig. 2 Excel files used for each plots, uncropped gel pictures.

Source Data Extended Data Fig. 3 Excel files used for each plot.

Source Data Extended Data Fig. 4 Excel files used for each plot.

Source Data Extended Data Fig. 5 Excel files used for each plot.

Source Data Extended Data Fig. 6 Excel files used for each plot.

Source Data Extended Data Fig. 8 Excel files used for each plot.

Source Data Extended Data Fig. 9 Excel files used for each plot.

Source Data Extended Data Fig. 10 Excel files used for each plot.

## Data Availability

The database used in this study is ChEMBL Database Release 32, 2023. All data are available within the Article and its Supplementary Information. All raw data are available via Dryad at 10.5061/dryad.fxpnvx10r (ref. ^40^). Source data are provided with this paper.

## Code availability

All code described in the manuscript is either provided in the Supplementary Information or available via the GitHub repository of M.S.S. at https://github.com/SigmanGroup/small-molecule-partitioning. Code for subsample correlation and differential enrichment analyses is available via the GitLab repository of M.K.R. at https://git.biohpc.swmed.edu/rosen-lab/small-molecule-properties-define-partitioning-into-biomolecular-condensates.
