# Supplementary material for: Small-molecule properties define partitioning into biomolecular condensates
Source: Nat Chem. 2024 Sep 13;16(11):1794–802. doi: 10.1038/s41557-024-01630-w (PMC11527791; doi:10.1038/s41557-024-01630-w)
Supplement: Supplementary file 1 — Supplementary Methods, Figs. 1–3, Table 1 and Methods-only references. [file 41557_2024_1630_MOESM1_ESM.pdf]

nature chemistry

Supplementary information https://doi.org/10.1038/s41557-024-01630-w

# Small-molecule properties define partitioning into biomolecular condensates

In the format provided by the authors and unedited

## Table of contents

1. Methods……………………………………………….……………… Page 2
2. Supplementary Figure 1............................................................. Page 30
3. Supplementary Figure 2............................................................. Page 31
4. Supplementary Figure 3............................................................. Page 35
5. Table S1......................................................................................... Page 36
6. Method-only references…………………………………........ Page 40

## Methods

### Genes, Plasmids, and DNA

polyPRM, polySH3, polySUMO, polySIM, Dhh1, GFP-Dhh1, and human cGAS were described previously[1-4]. For detailed information, see supporting information. Sequences of proteins and DNA used in this study are listed in Table S1. All the genes, except human cGAS, were cloned into a modified pMAL plasmid (pMTTH) containing TEV-cleavable N-terminal MBP and C-terminal $His_6$ tags. Full-length human cGAS was cloned into a pSUMO bacterial expression vector containing Ulp1-cleavable N-terminal $His_6$ and SUMO tags. Sense and antisense single stranded DNAs (45bp) of a cGAS immunostimulatory DNA (ISD)[3] were purchased from integrated DNA technologies. Double-stranded ISD was generated by annealing the single stranded DNA oligonucleotides in 25 mM HEPES-NaOH pH 7.5, 50 mM NaCl ramping temperature from 95 °C to 25 °C at 1 °C/min[3]. Annealing efficiencies were > 99% as assessed by HPLC.

### Protein Expression, Purification, and Labeling

All proteins were expressed and purified from *E. coli* strain BL21 DE3T1R. Unless otherwise specified, all the purification steps were carried out at 4 °C. All proteins, except Dhh1, GFP-Dhh1, and cGAS, were purified using a similar protocol[1,2]. Transformed bacteria were grown to $OD_{600}$ of 0.6-0.8 and then induced with 1 mM IPTG at 18 °C for 16 hours. Cells were collected by centrifugation (4,700 x g, 30 minutes) and the pellet was resuspended in 50 mM Tris pH 8, 150 mM NaCl, 10 mM imidazole, and 5 mM β-mercaptoethanol (BME) with protease inhibitors. Cells were lysed using a cell disruptor (Emulsiflex-C5, Avestin), and lysates were cleared by centrifugation (45,000 x g, 45 minutes). Proteins were affinity-purified with Ni-NTA Agarose Resin (Qiagen), followed by Amylose Resin (NEB). The amylose eluate was digested with TEV

protease (~1:100) overnight at 4 °C, filtered (0.22 µm) and loaded onto anion/cation exchange resin (Source15Q or Source 15S, GE Healthcare), then eluted with a linear gradient of NaCl (50–400 mM) in 50 mM Tris pH 8.0, 1 mM DTT and 1 mM EDTA. Protein-containing fractions were collected, concentrated, filtered, and further purified using size exclusion chromatography (Superdex 75 or 200, GE Healthcare) in 25 mM HEPES-NaOH pH 7.4, 150 mM NaCl, and 1 mM DTT buffer. After size exclusion chromatography, protein-containing fractions were concentrated by ultrafiltration (Amicon centricon) with 3k (polyPRM and polySIM) and 10k (polySH3 and polySUMO) molecular weight cutoffs. Single-use aliquots were flash frozen in liquid nitrogen and stored at -80 °C.

For Dhh1/GFP-Dhh1, the cell pellet was resuspended in lysis buffer (50 mM Tris pH 8, 500 mM NaCl, 10 mM imidazole, 5 mM BME with protease inhibitors). Cells were lysed using a cell disruptor (Emulsiflex-C5, Avestin), and lysates were cleared by centrifugation (45,000 x g, 45 minutes). Cleared lysate was incubated for 1 hour at 4 °C with Ni-NTA agarose resin (Bio-Rad) equilibrated with lysis buffer. Proteins were eluted by gravity chromatography using the following buffers. wash 1: 50 mM Tris pH 8, 500 mM NaCl, 10 mM imidazole, 5 mM BME; wash 2: 50 mM Tris pH 8, 100mM ATP, 2.5 M NaCl, 10 mM imidazole, and 5 mM BME; wash 3: 50 mM Tris pH 8, 10 mM imidazole, and 5 mM BME; elution: 50 mM Tris pH 8, 500 mM NaCl, 300 mM imidazole, and 5 mM BME. The eluate was added to amylose resin (New England Biolabs) and incubated for 30 minutes. Proteins were eluted by gravity chromatography using the following buffers. Amylose wash 1: 50 mM Tris pH 8, 150 mM NaCl, and 5 mM BME; amylose elution: 50 mM Tris pH 8, 150 mM NaCl, 5 mM BME, and 50 mM maltose. The amylose eluate was diluted threefold into 50 mM Tris pH 8, 5 mM BME buffer for a final concentration of 50 mM NaCl and filtered through a 0.22 µm Whatman filter (GE Healthcare). Filtrate was loaded onto a Source15Q

ion-exchange column and eluted with a linear gradient of NaCl (50 – 500 mM) in 50 mM Tris pH 8.0, 1 mM DTT and 1 mM EDTA. Fractions containing the desired protein were collected, concentrated, filtered, and loaded onto a SD200 size exclusion column equilibrated with 25mM HEPES-NaOH pH 7.4, 150 mM NaCl, and 1 mM DTT buffer. After size exclusion chromatography, protein-containing fractions were concentrated by ultrafiltration (Amicon centricon) with 10k molecular weight cutoffs. Single-use aliquots were flash frozen in liquid nitrogen and stored at -80 °C.

For full-length human cGAS (hcGAS-FL) purification[3], the cell pellet was resuspended in lysis buffer (50 mM Tris pH 8, 500 mM NaCl, 10 mM imidazole, 5 mM BME with protease inhibitors). Cells were lysed using a cell disruptor (Emulsiflex-C5, Avestin), and lysates were cleared by centrifugation (45,000 x g, 45 minutes). Cleared lysate was incubated with Ni-NTA agarose resin (Bio-Rad) equilibrated with lysis buffer for 1 hour at 4 °C with circular rotation in 50 mL conical tubes. Proteins were eluted by gravity chromatography using the following buffers. wash 1: 50 mM Tris pH 8, 500 mM NaCl, 10 mM imidazole, 5 mM BME; wash 2: 50 mM Tris pH 8, 100mM ATP, 2.5 M NaCl, 10 mM imidazole, and 5 mM BME; wash 3: 50 mM Tris pH 8, 10 mM imidazole, and 5 mM BME; elution: 50 mM Tris pH 8, 300 mM NaCl, 300 mM imidazole, and 5 mM BME. The eluate was digested with Ulp (~1:100) overnight at 4 °C, filtered through a 0.22 µm Whatman filter (GE Healthcare) and loaded onto a HiTrap Heparin column (GE Healthcare), then eluted with a linear gradient of NaCl (50–500 mM) in 50 mM Tris pH 8.0, 1 mM DTT and 1 mM EDTA. Protein-containing fractions were collected, concentrated, filtered, and further purified using size exclusion chromatography using an SD200 size exclusion column (GE Healthcare) in 25 mM HEPES-NaOH, pH 7.4, and 150 mM NaCl. After size exclusion chromatography, protein-containing fractions were concentrated by ultrafiltration (Amicon

centricon) with 10k molecular weight cutoffs. Single-use aliquots were flash frozen in liquid nitrogen and stored at -80 °C.

For all proteins, purity was assessed at each step of purification using SDS-PAGE. For experiments requiring protein fluorescence (protein PC value and droplet volume measurements using confocal fluorescence microscopy), all proteins except Dhh1 (we used GFP-Dhh1 for protein fluorescence) were labeled using maleimide-conjugated Alexa 488 dye (Life Technologies) following the manufacturer's protocol. After labeling, proteins were separated from free dye on an SD200 size exclusion column (GE Healthcare) in 25 mM HEPES-NaOH, pH 7.4, and 150 mM NaCl, and concentrated by ultrafiltration (Amicon centricon, 10k molecular weight cutoff). As assessed by UV-Vis spectrophotometry, all proteins were labeled at approximately 95%. Single-use aliquots were flash frozen in liquid nitrogen and stored at -80 °C.

**Metabolites, drugs, and small molecule fluorophores**

The metabolite library of 200 compounds (Table S2) used in this study is a calibration standard for targeted metabolomics, which covers most major metabolic pathways, including glycolysis, the tricarboxylic acid cycle, the pentose-phosphate pathway, and metabolism of amino acids and nucleotides. A metabolite stock (50 µM of each compound) was prepared in MS grade water (Sigma-Aldrich) and used in partitioning experiments to give a final concentration of 2 µM for each compound. The drug library used is the Prestwick Chemical Library®:1520 FDA-approved & EMA-approved drugs (Prestwick chemical libraries (PCL1520.10-100-96G, Table S3). We purchased drugs as individual 10 mM stock solutions in DMSO. We mixed compounds to prepare sub-libraries of ~300 molecules of unique molecular weight (30 µM of each drug in MS grade water/DMSO 10/90% by volume. For the drug partitioning experiments, the sub-libraries

were diluted 30-fold into the reaction mixture, producing a final concentration of ~1 µM of each compound and ~3% DMSO. Using a ChemiDoc XRS+ system (Bio-Rad) at different excitation wavelengths, 488 nm, 546 nm, and 647 nm, we identified 34 fluorescent molecules in the drug library (Table S4). We prepared individual working stock solutions of these molecules (200 µM in MS grade water/DMSO, 98/2 % by volume) for use in partitioning experiments using confocal fluorescence microscopy. To optimize our methods to quantify small molecule partitioning into biomolecular condensates, including extraction of compounds from droplet and bulk samples (see below), we used commonly available fluorophores, such as FITC, Rhodamine, Alexa488, etc. (Table S4). We prepared individual working stock solutions of these fluorophores (100 µM in MS grade water/DMSO, 99/1 % by volume) for use in partitioning experiments using confocal fluorescence microscopy and MS.

**Microscopy plate preparation**

Microscopy experiments were carried out in 384-well glass bottom microwell plates (Brooks Life Science Systems: MGB101-1-2-LG-L). Prior to use, plates were washed with 5% Hellmanex at 37°C for 4 h and then extensively with MilliQ water. Glass was etched with 1 M NaOH for 1 h at room temperature, washed extensively with MilliQ water, and then treated overnight (≥16 h) at room temperature with 25 mg/mL 5K mPEG-silane (PEGWorks) in 95% Ethanol. The plate was washed once with 95% ethanol, extensively with MilliQ water, and then dried in a chemical hood for 3-4 h. PEGylated microscopy plates were sealed with adhesive PCR plate foil (Thermo). Immediately prior to use, foil was cut above individual wells and both plastic and PEGylated glass were passivated by incubation with freshly prepared 10 mg/mL BSA for 30 min. Wells were rinsed once with MilliQ water, followed by buffer (25mM HEPES-NaOH (pH 7.4) with 150 mM NaCl) to remove excess BSA, and microscopy samples (50-60 µL) were

immediately added. Desiccation of microscopy samples was minimized following transfer to the plate by sealing with transparent sealant tape.

**Measurement of protein PC values and droplet volume fraction by fluorescence microscopy**

We measured protein partition coefficient (PC) values and calculated droplet volume fraction using confocal fluorescence microscopy with 1% Alexa-488 labeled polySUMO, polySH3, and cGAS, or 1% GFP-labeled Dhh1 in 25mM HEPES-NaOH (pH 7.4), 150 mM NaCl buffer. We used the following concentrations of each species: 5 µM polySUMO (module concentration of 50 µM) and 5 µM polySIM (module concentration of 50 µM); 10 µM polySH3 (module concentration of 50 µM) and 10 µM (polyPRM (module concentration of 50 µM); 5 µM MBP-Dhh1 and 0.2 µM TEV-protease; 2 µM cGAS and 2 µM 45bp DNA. The scaffold mixtures were incubated for 1 hour (cGAS-DNA), 4 hours (SUMO/SIM), 12 hours (SH3/PRM), or 20 hours (Dhh1) at room temperature. After incubation, we acquired images using a 20x air objective (for SUMO/SIM and SH3/PRM) or 60x oil immersion objective (for Dhh1 and cGAS-DNA) on a Leica SP8 Laser Scanning Confocal Microscope.

All images were corrected for dark counts and non-uniform illumination prior to analysis. To avoid dilution effects of the microscope point spread function (PSF) on intensities of smaller condensates, for partition coefficient and droplet volume measurements, we only analyzed droplets with x-y diameter >2-fold larger than the z-dimension PSF. Intensities from all included regions of droplets or bulk phases were separately averaged and used to calculate concentrations in the two phases using intensity vs. concentration standard curves. Standard curve solutions were prepared alongside experimental samples using GFP-Dhh1 or Alexa 488-labeled molecules (polySUMO, polySH3, and cGAS) in the corresponding experimental buffer (25mM HEPES-

NaOH (pH 7.4), 150 mM NaCl) supplemented with 0.1-0.2 mg/mL BSA to prevent fluorophore adsorption to surfaces. Partition coefficients were determined from the bulk and droplet concentrations.

$$\text{Partition coefficient} = \frac{Droplet\ concentration}{Bulk\ concentration}$$

Droplet volume fraction was determined from the total, droplet and bulk concentrations by [5]:

$$\text{Droplet volume fraction} = \frac{(Total\ concentration - Bulk\ concentration)}{(Droplet\ concentration - Bulk\ concentration)}$$

Note that Extended Data Figure 2 illustrates that, within experimental error (~25% in biological replicates), droplet volume fractions do not appear to change upon addition of the drug libraries.

**Microscopy assay to measure partitioning of fluorescent small molecules**

To measure the partition coefficients of fluorescent small molecules using microscopy, we used the following concentrations of unlabeled scaffold macromolecules mixed with individual small molecules (100 nM-1 µM) in 25 mM HEPES-NaOH (pH 7.4), 150 mM NaCl buffer: 5 µM polySUMO (module concentration of 50 µM) and 5 µM polySIM (module concentration of 50 µM); 10 µM polySH3 (module concentration of 50 µM) and 10 µM polyPRM (module concentration of 50 µM); 5 µM MBP-Dhh1 and 0.2 µM TEV-protease; 2 µM cGAS and 2 µM 45bp DNA. The macromolecules were added to prepared 384-well plates first, followed by the fluorescent small molecules; mixtures were incubated for 1 hour (cGAS-DNA), 4 hours (SUMO/SIM), 12 hours (SH3/PRM), or 20 hours (Dhh1) at room temperature. After incubation, we acquired images using a 20x air objective (for SUMO/SIM and SH3/PRM) or 60x oil immersion objective (for Dhh1 and cGAS-DNA) on a Leica SP8 Laser Scanning Confocal

Microscope. Fluorescence intensity (*I*) from the droplet and bulk phases were used to measure the partition coefficients. All images were corrected for dark counts and non-uniform illumination prior to analysis. As a background control, an equal volume of DMSO was added to the unlabeled scaffold mixture, and measured values in the droplet and solution phases were used in calculation of partition coefficients according to:

$$\text{Partition coefficient} = \frac{(I_{\,Droplet(drug)} - I_{\,Droplet(DMSO)})}{(I_{\,bulk(drug)} - I_{\,bulk(DMSO)})}$$

**Mass spectrometry assay to measure partitioning of small molecules**

***Extraction of small molecules from droplet and bulk samples***

The mass spectrometry (MS) assay required efficient extraction of small molecules from condensates and bulk solutions. To optimize this process[6], we first measured partitioning of small molecule fluorophores (Table S4) into the SH3PRM condensate using confocal fluorescence microscopy as a gold standard, as described above. We then compared this value to those measured by fluorescence spectroscopy and MS, which both required extraction of the dye from droplet and bulk solutions. To produce these samples, we incubated SH3PRM condensates with FITC, and then separated droplets from bulk by centrifugation as detailed in the following section. To extract the dye from the samples we first digested the protein scaffolds with trypsin, varying time (1-12 hours), temperature (25 °C or 37 °C), and enzyme concentration (0.01-5 µg/µL). We used methanol (80:20 v/v solvent:sample) to quench the reaction and precipitate the digested proteins at different temperatures (4 °C, -20 °C, -80 °C) and for different durations (4-24 hours). For each combination of treatments, we quantified the amount of dye present in the droplet and bulk fractions by fluorescence spectroscopy ($\lambda_{ex}$ = 488 nm, $\lambda_{em}$ = 495-600 nm) through a standard curve.

In parallel, we also measured the amount of extracted FITC by MS as detailed below. Processing conditions were optimized to reproducibly yield identical PC values by all three methods (fluorescence microscopy, fluorescence spectroscopy and MS).

We further examined the efficiency of extraction of compounds from the protein samples in three ways:

1. We incubated the SH3PRM condensate with 1 µM FITC, and processed the condensate sample as described for mass spectrometry below. We assessed the efficiency of the post-digestion methanol extraction by extracting multiple times, and measuring the fluorescence of the recovered material. As shown in Supplementary Figure 3A, >95% of the dye is removed from the pellet in the first step, with much smaller amounts coming out later.
2. We examined extraction efficiency (quantified as extraction 1 / (extraction 1 + extraction 2)) more broadly using the SH3PRM and Dhh1 condensates and one of our 300-compound sublibraries. After processing, we extracted the tryptic digest pellets of the condensate and bulk samples twice each with 80% methanol, and used mass spectrometry to compare them. As shown in Supplementary Figure 3B, for both condensate and bulk samples, for 80-85 % of compounds in the sublibrary >70% of material was recovered in the first extraction, and only 5-8 % had Extraction 2 > Extraction 1. Moreover, as shown in Supplementary Figure 3C, the PC values determined from the sum of Extraction 1 and Extraction 2 (of both condensate and bulk) are very similar to those determined from Extraction 1 alone, indicating that less extraction for some compounds does not alter the measured PC values.
3. We modified our extraction protocol to use 80% acetonitrile, which is more hydrophobic than 80% methanol and should be better at removing highly-partitioning (hydrophobic) compounds from the tryptic digest pellet. We applied this protocol to the same condensate

and library as in point 2. As shown in Supplementary Figure 3D nearly all of the compounds had very similar PC values whether extraction was performed with acetonitrile or methanol.

Thus, we conclude that the methanol extraction step in our protocol is efficient and enables accurate measurement of PC values by mass spectrometry.

Final conditions used in the assays are described in the sections below. We note that tryptic digestion prior to methanol precipitation was essential to achieving complete extraction of dye from the condensate samples.

***Sample preparation for condensates reconstituted in simple buffers***

For small molecule partitioning experiments using mass spectrometry (MS) we first mixed macromolecular scaffolds and then added a final concentration of 2 µM of each metabolite (total of 200 metabolites, giving total metabolite concentration of 400 µM) or 1 µM of each drug molecule (total of 300 drug molecules/sub-library, giving total drug concentration of 300 µM, with a final DMSO concentration of 3% v/v) in 25mM HEPES-NaOH (pH 7.4), 150 mM NaCl buffer. Scaffolds were used at the following concentration: 5 µM polySUMO (module concentration of 50 µM) and 5 µM polySIM (module concentration of 50 µM); 10 µM polySH3 (module concentration of 50 µM) and 10 µM polyPRM (module concentration of 50 µM); 5 µM MBP-Dhh1 and 0.2 µM TEV-protease; 2 µM cGAS and 2 µM 45bp DNA. In each case macromolecules were mixed first, followed by small molecules. Mixtures were incubated for 1 hour (cGASDNA), 4 hours (SUMOSIM), 12 hours (SH3PRM), or 20 hours (Dhh1) at room temperature. The total volume of each reaction was 1000 µL. Experiments were replicated twice and 8-9 times for drugs and metabolites, respectively. We also prepared the following control samples for each

condensate: (1) macromolecules without any added small molecules, (2) small molecules without any macromolecules, and (3) single macromolecule component of condensates with small molecules (e.g., polySUMO or polySIM alone with small molecules; in all such samples phase separation did not occur). After incubation, samples were centrifuged at 14,000 g for 30 min using a temperature-controlled centrifuge carefully maintained at 22 °C. We note that the cGASDNA[3] and Dhh1[7] condensates mature from liquids to more solid-like states over time, and that under the conditions and incubation times here both show only modest recovery in FRAP experiments (Supplementary Figure 1 and Extended Data Figure 2I). It is possible that the partitioning profiles could change between liquid- and solid-like states of a condensate, a question we plan to address in future studies. Also, we have not accounted here for potential irreversible changes to condensates that may have occurred during centrifugation. Nevertheless, the reasonable correlations between the mass spectrometry and fluorescence measures of partitioning for the majority of fluorescent compounds suggest that any such changes are not likely to be large or systematic.

After resting on the bench for ~5 minutes, the supernatant was transferred carefully and as completely as possible to a new 1.5 mL centrifuge tube, leaving the condensate pellet (typically ~10 µL, for 1000 µL total reaction volume) in the original tube. Based on the droplet volume measurements (see above), we transferred an equal droplet volume of the supernatant into a new micro centrifuge tube. The supernatant and pellet fractions were each mixed with trypsin (10 µL, from 0.1 µg/µL stock concentration MS-grade, Porcine; Fisher Scientific) prepared in 25mM HEPES-NaOH (pH 7.4), 150 mM NaCl buffer in MS grade water, and incubated at 37 °C for 12 h with gentle shaking. After incubation, the reactions were mixed well by pipetting, and 80 µL of MS grade methanol pre-chilled to -80 °C was added (to give 80% v/v methanol). Samples were

vortexed for 30 s and incubated at -80 °C for 12 hours. After incubation, samples were vortexed again for 30 s and centrifuged at 20,000 g at 22 °C for 45 minutes. After centrifugation, supernatant fractions containing small molecules were carefully removed and kept at -80°C prior to MS analysis.

***Sample preparation for condensates reconstituted in U2OS cell lysates, and Xenopus extracts***

To generate SUMOSIM condensates in U2OS cell lysates, we modified a protocol used for biomimetic reconstitutions of stress granules and nucleoli[8]. U2OS cells (HTB-96) were cultured in DMEM ((HyClone) supplemented with 10% FBS (HyClone; SH30071.03 and SH30396.03) and maintained at 37°C in a humidified incubator with 5% $CO_2$. Cells were grown to 100% confluency in 10-cm cell culture–treated dishes, the medium was aspirated, and cells were washed with PBS. Cells were detached by scraping in 5 ml PBS and recovered by centrifugation at 500 *g* for 3-5 min. Buffer was carefully removed by aspiration and cell pellets were stored at −80°C. To prepare lysate, pellets (10-15 µL volume) were resuspended in 250 µL cell lysis buffer (25 mM HEPES-NaOH (pH 7.4), 100 mM NaCl with protease inhibitors (Roche 11836170001)), and pipetted 5-10 times using a 200 µL tip to produce a visibly homogenous solution, followed by incubation on ice for 10 min. The suspension was freeze-thawed five times by alternating between liquid nitrogen (15 sec) and a 37° water bath (2 min), and finally pipetted twice using a 200 µL tip. The lysate was cleared by centrifugation at 20,000 g for 5 min at 4 °C. The supernatant was transferred to a fresh tube and used within 1-2 hours. Total protein concentration in the cleared lysate was estimated as 4 mg/ml using the Bradford assay (Bio-Rad, Cat# 5000002). To generate condensates in this system, unlabeled polySUMO and polySIM were added to cleared cell lysate to final concentrations of 10 µM, followed by 1 µM each drug molecule (10 µL of 30 µM stock of 300 drug molecules/sub-library), giving a final assay volume of 280 µL (final composition of 3%

v/v DMSO). We also prepared control samples containing drug molecules in lysis buffer or cell lysate alone (without polySUMO or polySIM proteins). After drug addition, mixtures were incubated at room temperature for 4 hours, and droplet and solution phases were separated by centrifugation at 2000 g for 15 min at 22°C. Note that these conditions were optimized to minimize background sedimentation of drugs with the lysate only control, while still recovering most of condensates. The supernatant was transferred carefully and as completely as possible to a new 1.5 mL centrifuge tube, leaving the condensate pellet (typically ~5 µL) in the original tube. Based on the droplet volume measurements (see above, and Extended Data Figure 2 illustrating that the amount of scaffold proteins in droplet and bulk solutions do not change within experimental error upon addition of the drug libraries), we transferred an equal droplet volume of the supernatant into a new centrifuge tube. The supernatant and droplet fractions were processed and prepared for MS as described above for condensates in simple buffers.

Xenopus oocyte extracts were prepared using a standard protocol[9] and flash frozen at -80 °C. To minimize background sedimentation of drugs in the absence of condensates, we clarified the raw extract by ultracentrifugation at 100,000 g for 1 hour at 4 °C prior to use. Total protein concentration in the cleared extract was estimated as ~80 mg/ml using the Bradford assay (Bio-Rad, Cat# 5000002). Assays containing SUMOSIM condensates and drugs were generated and processed as for the U2OS cell lysates.

***Targeted Metabolomics approach to quantify metabolites***

We used a previously described protocol to quantify metabolites extracted from the different samples[10]. LC-MS/MS mass spectrometric analyses were performed on a Sciex QTRAP 6500+ mass spectrometer equipped with an electrospray ion (ESI) source. The ESI source was

used in both positive and negative ion modes, configured as follows:  Ion Source Gas 1 (Gas 1), 40psi; Ion Source Gas 2 (Gas 2), 35 psi; curtain gas (CUR), 50 psi in the negative polarity mode and 45 psi in the positive polarity mode; source temperature, 550 °C; and ion spray voltage (IS), +4800 V(+) and −4000 V (−). The mass spectrometer was coupled to a Shimadzu HPLC (Nexera X2 LC-30AD). The system is controlled by Analyst 1.7.2 software.

Hydrophilic interaction chromatography was performed using a SeQuant® ZIC®-pHILIC 5 µm polymeric 150 × 2.1 mm PEEK coated HPLC column (Millipore Sigma, USA). The column temperature, sample injection volume, and flow rate were 45 °C, 5 µL, and 0.15 mL/min respectively. HPLC conditions were as follows: Solvent A: 20 mM ammonium carbonate including 0.1% Ammonium hydroxide. Solvent B: Acetonitrile. Gradient pattern was 0 min: 80% B, 20 min: 20% B, 20.5 min 80% B, 34 min: 80% B. The mass spectrometer was equipped with a switching valve such that the column effluent between 0-2 min and 18-34 min was delivered to the waste to avoid interfering ions entering the ESI source in the negative polarity mode. In the positive polarity mode, the column effluent between 0-1min and 18-34 min was delivered to waste. All targeted metabolites were eluted from the column between 2-15 minutes. Data were processed using SCIEX OS 2.1 software with relative quantification based on the peak area of each metabolite.

***Untargeted metabolomic approach to quantify drugs and other small molecules***

To detect and quantify drugs and other small molecules such as fluorophores, we employed a standard untargeted metabolomic approach[11]. Mass spectrometric analyses were performed on a Sciex TripleTOF 6600 system (AB SCIEX, Framingham, MA, USA) equipped with electrospray ionization (ESI), atmospheric pressure chemical ionization (APCI) sources and calibrant delivery

system (CDS). The electrospray ionization (ESI) source used in the positive and negative ionization mode and configured as follows: Ion Source Gas 1 (Gas 1), 50psi; Ion Source Gas 2 (Gas 2), 45 psi; curtain gas flow, 35 psi; source temperature, 550 °C; and ion spray voltage floating, +5500 V(+) and −4500 V (−). TOF-MS mode (Full scan) and Information Dependent Acquisition (IDA) mode (Product Ion scan) were utilized to collect MS and MS/MS data, respectively. For TOF-MS scans, the mass range was from m/z 70 to 1000 and for Product Ion scans, the mass range was from m/z 30 to 1000. The collision energy (CE) was set at 30 V (+) or -30 V (-) and collision energy spread (CES) was ±15 V. The accumulation time was 0.25 seconds for TOF-MS scans and 0.05 seconds for product ion scans. The instrument was automatically calibrated for mass accuracy (<5 ppm), including MS1 scan and MS/MS scan, every five samples using APCI calibration solution. The mass spectrometer was coupled to a Shimadzu HPLC (Nexera X2 LC-30AD). The system was controlled by Analyst TF 1.8.1 software (Sciex).

Reverse phase chromatography was performed using an ACE 3 C18-PFP 150 x 4.6 mm HPLC column (Mac-Mod, USA). The column temperature, sample injection volume, and flow rate were 30°C, 10 µL, and 0.5 mL/min respectively. The HPLC conditions were as follows: Solvent A: Water with 0.1% Formic Acid (v/v), LC/MS grade and Solvent B: Acetonitrile with 0.1% Formic Acid (v/v), LC/MS grade. Gradient condition was 0-2 min: 5% B, 5-16 min: 90% B, 17 min 5% B, 30 min: 5% B. Compounds eluted in a linear gradient of 5% to 90% B over 14 minutes were used for the analysis. Data were processed using SCIEX OS 2.1 software with relative quantification based on the peak area of each small molecule, with signal intensities in the linear range of the detector ($10^3$ – $10^8$ counts/second).

***Mass spectrometry data analysis and PC calculation***

We initially filtered the mass spectrometry data for quality in several respects, taking a conservative approach that eliminated roughly 300-400 compounds in the library for each condensate (Table S6):

1. If signal from either the condensate or bulk sample was below the noise threshold of the mass spectrometer ($10^3$counts), the compound was excluded. Compounds were also excluded if either signal was > $10^9$, the maximum of the linear range of the detector.

2. If the mass error in the mass spectrometry peak detection for either the condensate or bulk sample was > ±5 ppm, the compound was also excluded.

3. Compounds that did not separate well on the chromatography column preceding the mass spectrometer injector were excluded.

4. Compounds with molecular weight greater than 1000 Da were excluded.

5. Metabolites, such as amino acids, that were present in significant amounts in buffers or purified proteins, or were produced during protease digestion of proteins were excluded.

6. Nucleotide phosphates, such as ATP or GTP, that were unstable during processing or in the mass spectrometer were excluded.

With these filters in place, we analyzed the remaining data from the four condensates to account for potential precipitation of compounds in the buffers. We used different controls based on the type of condensate system. As controls for the two-component condensates (SH3PRM,

SUMOSIM, cGASDNA), we examined compounds that sedimented upon incubation with only the individual macromolecules (no observable LLPS). As control for the Dhh1 condensate, we examined compounds sedimenting in buffer alone. In all controls, following identical centrifugation and extraction steps as in the condensate incubations, we measured the amounts of material in the pellet fraction of the control incubation (which is where artifactual precipitation would manifest). The mass spectrometry data from these control measurements are reported in Supplemental Table S5 and S7.

We then processed the data according to the ratio of integrated mass spectrometry peak intensities in the control and condensate incubations. For the two-component condensates we averaged the ratio values of each single-component control to produce a final value, $R_{control}$ (we also use this same term for the ratio calculated from buffer control values in the case of Dhh1). $R_{control}$ values are listed in Supplemental Table S7.

Compounds with $R_{control} > 0.5$ (i.e., where the amount sedimented in the control incubation was greater than half the amount sedimented with the condensate) were deemed to be overly influenced by precipitation, and were discarded from the analysis. As shown in Supplemental Table S6, this resulted in elimination of 1.2 - 4.5 % of compounds in the different condensates.

For all remaining compounds we calculated the PC value using the following equation, which corrects for the amount of material that co-sediments on centrifugation due to insolubility along with the material that is partitioned into the condensate:

$$PC = \frac{Peak\ area_{pellet}(condensate) - \ Peak\ area_{pellet}(control)}{Peak\ area_{supernatant}(condensate)}$$

We note that with the $R_{control}$ < 0.5 cutoff, this correction changes PC values by at most 2-fold. Moreover, since the large majority of compounds have $R_{control}$ < 0.1 (83 - 95 %, Supplemental Table S6), most corrections are < 10%, which is less than the measurement error in the mass spectrometry.

We used an analogous procedure with the U2OS lysate and Xenopus extract solutions, where control samples consisted of the compounds in lysate/extract, without SUMOSIM addition. We observed that in general $R_{control}$ values for the compounds are larger in the cell lysate and X. laevis extract than in the buffer-alone system. Compounds with $R_{control}$ > 0.5 were eliminated from further consideration, and PC was calculated according to:

$$PC = \frac{Peak\ area_{pellet}(SUMOSIM + lysate) - \ Peak\ area_{pellet}(lysate\ alone)}{Peak\ area_{supernatant}(SUMOSIM + lysate)}$$

As shown in Supplemental Table S6, more compounds were eliminated in the lysate/extract systems than with the purified proteins, and the corrections involved a larger fraction of the molecules.

**Isothermal Titration Calorimetry**

ITC measurements were performed at 25 °C and 35 °C using a MicroCal PEAQ-ITC calorimeter (Malvern Panalytical). Purified polySUMO and polySIM were buffer exchanged by

size exclusion chromatography on a Superdex200 column with a mobile phase buffer of 25 mM HEPES buffer (pH 7.4) and 150 mM NaCl. Protein concentrations were measured using UV-Vis spectrophotometry with calculated extinction coefficients, $\varepsilon_{280nm,polySUMO}$ = 27,390 $M^{-1}cm^{-1}$ and $\varepsilon_{280nm,polySIM}$ = 6,990 $M^{-1}cm^{-1}$. Proteins were flash frozen in liquid nitrogen and stored at −80 °C. Immediately prior to measurement, proteins were thawed and diluted into the same buffer and mixed at a final concentration of 2 µM polySUMO and 2 µM polySIM (20 µM module concentrations), which is below the LLPS threshold. DMSO was also added to a concentration of 2% v/v, to match that of the drug solution in the syringe. For each data point, 1.9 µL of 200 µM drug in the same buffer were injected into 0.3 ml of protein every 120 s. Twenty injections were performed per experiment. We also acquired analogous control thermograms where drug molecules were injected into buffer, or where buffer was injected into protein. The latter did not produce significant heat changes and was not used for further correction of data. However, injection of some drug molecules into buffer produced significant heat changes. In these cases, the control thermogram was subtracted from the drug + protein thermograms prior to analysis. Data for raw ITC and thermodynamic curves were analyzed using Microcal PEAQ-ITC software and plotted using SigmaPlot software.

**PRODAN-dye based condensate polarity measurements**

We used the solvatochromatic dye, 1-[6-(Dimethylamino)naphthalen-2-yl]propan-1-one (PRODAN), to characterize the polarity of the four biomolecular condensates studied here. We used confocal fluorescence microscopy (Leica SP8, $\lambda_{ex}$ 405 nm) to collect 5 nm wide emission spectral window measurements between 420 nm to 650 nm for the droplet and bulk phases. The wavelength of maximum emission intensity was estimated visually and could be compared to those

obtained for PRODAN dissolved in pure solvents of different polarities, such as acetonitrile, DMSO, ethanol, methanol, and water.

**Sub sample correlation analysis**

As a qualitative depiction of the range of PC values at which correlations in PCs between the different condensate systems emerges, we developed an algorithm to characterize how correlations vary across different subsamples of the PC data. We subsample the data in multiple overlapping windows with a normally distributed bias, while varying the mean and standard deviation of the biasing normal distributions. We calculate Pearson correlation coefficients for the data in each window, then display the correlation coefficients as a raster image where x-axis position represents the center of the sampling window (i.e., the mean of the normal distribution that biases the sampling), and the y-axis position represents the size of the sampling window (i.e., the standard deviation). Note that if only two PC value pairs are sampled, the correlation coefficient will be 1 regardless of data values and is thus uninformative and we do not show them on our plots. The algorithm in pseudocode is below and R code[12] implementing the algorithm is provided as Supplementary Software.

**input: PC data vector for condensate 1** $D_1$ (length $n$),

PC data vector for condensate 2 $D_2$ (length $n$),

$j$ center positions of sampling windows $m_1, \ldots, m_j$

$k$ sampling window sizes (standard deviations) $s_1, \ldots, s_k$,

$i$ sampling iterations $1, \ldots, i$

convergence tolerance $t$

**output:** **5 × (**$j$×$k$) matrix where the rows contain the 0, 0.25, 0.50, 0.75, and 1 quantiles of the sampled correlation coefficients and the columns correspond to each combination of $m$ and $s$

create empty vector *A # for quantiles of B*

**for each** $m$:

  **for each** $s$:

    create empty vector *B # for correlation coefficients*

    **for each** $i$:

      create empty vector *C # for samples*

      create empty vector *D # for fraction sampled*

      **while** tolerance not met:

        obtain sample $d$ from ($D_1$, $D_2$)

        obtain sample $x$ from Normal(0, $s$) distribution

        **if** $|d_1-m| \leq |x|$ and $d$ was not previously sampled:

          append $d$ to $C$

        append length($B$)/$n$ to $D$

        **if** last $t$ elements of $B$ are equal to the final element of $B$:

          end while loop

      append correlation coefficient of samples in C to B

    append vector ($q_0$, $q_{0.25}$, $q_{0.5}$, $q_{0.75}$, $q_1$) to $A$ (where the $q$s are quantiles of B)

We set $j$ and $k$ to 100, $i$ to 100, and $t$ to 100. Running in parallel on 6 28-core server nodes the calculations take approximately 5 h.

**Small Molecule Parameterization**

Low-energy 3D chemical structures of the small molecules used in this study (N = 1507) were generated from SMILES strings (Tables S2 and S3) with LigPrep[13] using the OPLS4 forcefield[14]. Of those molecules, 1304 had quantifiable partitioning in at least one condensate, however 54 molecules were dropped from modeling analysis due to errors calculating one or more QikProp descriptors The remaining 1250 molecules were desalted, and protonation and tautomer states and were corrected for a pH 7.0 solution using Epik[15]. The structures were encoded as 51 ADME features using QikProp[16]; input files containing the QikProp descriptors used to build the UMAP chemical space and statistical models are available on the Sigman Lab Github: https://github.com/SigmanGroup/small-molecule-partitioning. Mordred descriptors were computed and curated for the relevant statistical models from SMILES strings of the small molecules using an implementation of the Mordred package which is available on the Sigman Lab Github: https://github.com/SigmanGroup/Mordred-descriptors.

**Chemical Space Analysis and Clustering**

The QikProp descriptors were used as the input to generate a 2D UMAP chemical space[17]: an amenable UMAP embedding of the features was generated and the HSBCAN algorithm was applied to identify clusters[18]. HDBSCAN is an unsupervised clustering method that automatically identified and assigned molecules to 11 clusters. HDBSCAN also identified that 5% of small molecules were not clustered because they did not overlap in UMAP space with defined clusters. Code to reproduce the UMAP chemical space and associated clustering (including the specific algorithm parameters used in study) is available on the Sigman Lab Github: https://github.com/SigmanGroup/small-molecule-partitioning.

In addition to analyzing our chemical library alone, we also compared it to the much larger chemical space sampled by the ChEMBL database. ChEMBL contains 2.4 million small molecules with known bioactivity or drug-like properties[19]. Of the small molecules in the 32nd release of the ChEMBL library,[44] 2,084,720 included SMILES strings, which were accessed with the Therapeutic Drug Commons molgen function[45,20]. MACCS keys were computed due to their significantly faster calculation time using Open-source cheminformatics RDKit[21]. To generate a UMAP, the entire ChEMBL library was initially combined with our metabolite/drug library. However, analyzing all 2 million data points in the UMAP analysis was challenging due to scalability limitations. Therefore, a random subsample of approximately 10% of the compounds (~200,000 small molecules) was selected from the ChEMBL library for the UMAP analysis including the metabolites and FDA approved drugs in our study. As shown in Figure 1A, our collection of metabolites and drugs exhibited a well-dispersed distribution within the ChEMBL chemical space, indicating their representativeness of the bioactive molecules found in the ChEMBL database.

**Statistical Modeling**

The clustering analysis conducted on the QikProp UMAP space was leveraged to construct diverse and comprehensive training sets that sampled a broad spectrum of chemical space. For each of the 10 clusters identified through HBDSCAN analysis, 20% of the molecules were randomly selected as the external validation set. These molecules were withheld for all stages of model development and were only used to assess the performance of final models, which are described in the following sections.

An additional 20% of molecules were randomly designated as the test set, while the remaining 60% served as the training set for model development. Molecules not designated to a cluster (~5%) were all assigned to the training set. This division allowed for the comparison of different modeling strategies and facilitated the assessment of model performance on independent data. It is important to note that the selection of training, test, and validation sets was conducted in a fully blinded manner, ensuring unbiased evaluation. Due to the nature of the small molecule partitioning data, slight variations in the specific ratios for each data group were observed, as certain measurements were unobtainable for a small number of compounds with some condensates.

The descriptors were scaled by removing the mean and scaling to unit variance. Using training set statistics ($R^2$ and mean absolute error, MAE) as a benchmark, we assessed several tree-based statistical models, including random forest (RF) regressors, RF regressors with cost complexity pruning (CCP), and XGBoost models. Additionally, we compared the tree-based methods to a multilayer perceptron neural net. The neural net preformed worse than the tree-based methods, with the XGBoost model achieving the overall best performance (Table S10). For our initial survey of modeling algorithms, default parameters were used except as indicated in the attached code. RF and RF with CCP models were developed from ensembles of 1000 trees; for the models with CCP, the complexity parameter (cpp_alpha) was set to 0.001. Code to reproduce the statistical models in this study (including feature scaling and feature importance metrics) is available on the Sigman Lab Github: https://github.com/SigmanGroup/small-molecule-partitioning.

We also determined that the use of QikProp ADME-type descriptors resulted in better models than Mordred descriptors, which are more structurally based (see also next section).

Combining the two descriptor sets did not improve models, even if collinear descriptors ($R^2 > 0.9$) were eliminated or if descriptors were selecting selected using a *Z*-test mean comparison for excluded vs. included molecules using the statsmodels Python package (Table S9)[22]. Validation set molecules were not used in *Z*-test comparisons.

The XGBoost algorithm with QikProp features was implemented to make four separate but similar models of small molecules partitioning with SUMOSIM, SH3PRM, Dhh1, of cGASDNA condensates (Table S10, entries **1-4**). As described in the main text, an all condensate and an average LogPC model were also developed (Table S10, entries **5** and **6**). For the all-condensate model, the experimental condensate Prodan measurements were added to the small molecule feature set. The XGBoost algorithm parameters were tuned separately for each of the six models as described in Table S11. XGBoost parameters for the models of small molecule partitioning into SUMOSIM condensates in U2OS cell lysate and Xenopus *laevis* oocyte extract are also indicated in Table S11. For validation of the average logPC model, the training and test sets were pooled (N pooled = 784); the XGBoost model was retrained on the pooled without re-tuning of algorithm parameters and used to predict the average logPC for validation set molecules (N validation = 204).

## Comparison of ADME-based and structure-based descriptors

Molecular fingerprints are vector representations of molecular structures that encode information about which substructures are present in the molecule[21,23-25]. ECPF4 exhaustively expresses the presence of specific substructures around each atom in a molecule, resulting in many "circular" features that have been used to build structure-function models of small molecules. Molecular ACCess System (MACCS) keys are another type of molecular fingerprint that differ

from ECPF4 fingerprints by using fixed-length binary encoding. With 166 keys of features, each representing a specific chemical substructure or pattern, MACCS keys encode a range of molecular functional groups, including ring systems and other structural motifs commonly found in organic compounds. This provides a compact representation compared to the exhaustive algorithm used by ECPF4 fingerprints. To explore the suitability of different fingerprints for predictive models of small molecule partitioning, both ECFP4 and MACCS features were computed in RDKit[21], an open-source cheminformatics package from the small molecule SMILES strings. We then analyzed the SUMOSIM dataset using these structure-based features.

The ECFP4 model exhibited poorer performance compared to the similar QikProp model, with train and test $R^2$ of 0.89 and 0.15, respectively, and a logPC MAE of 0.75. Filtering out rare ECFP4 substructure features did not improve the fit of the XGBoost models for small molecule partitioning with SUMOSIM. Similarly, the MACCS fingerprint model had poorer statistics compared to the QikProp features, with train and test $R^2$ of 0.88 and 0.18, respectively, and a logPC MAE of 0.726. Filtering rare MACCS features also had minimal effect on the XGBoost models (Table S10)

To conclude the feature set analysis, a final comparison was conducted between the QikProp descriptors and the features calculated using the open-source package, Mordred[26]. The Mordred feature set encompasses a comprehensive collection of 1800 two- and three-dimensional structural and physicochemical descriptors. This comparison aimed to investigate whether combining both structural and physicochemical descriptors would enhance the performance of the models. However, it was found that the use of QikProp ADME descriptors yielded superior models compared to the Mordred descriptors, even after eliminating collinear descriptors ($R^2 > 0.9$) from the Mordred feature set.

The comparison of XGBoost models and the chemical space analysis using different feature sets strongly indicates that the QikProp features are uniquely suited for the objective of analyzing small molecule partitioning. These features demonstrate superior performance compared to the ECFP4 and MACCS keys, revealing that the physicochemical properties captured by QikProp play a more significant role in accurately describing and predicting small molecule partitioning behavior than structurally based features.

**Differential compound enrichment analysis**

To identify compounds that are differentially enriched when comparing different condensate systems, we adopted methods from differential gene expression analysis. Specifically, we used the linear modeling approach implemented in limma (version 3.54.2, Ritchie et al. 2015; R version 4.2.3, R Core Team 2023). Though originally developed for microarray analysis, the algorithm is appropriate for use with other types of continuous-valued data and has been previously applied to mass spectrometry data analysis[27]. Data was pre-processed as described above, except the two experimental replicates were not averaged, data for any compounds where any replicate had partition coefficient less than or equal to zero was discarded, and only compounds where data was available for all four condensates were included. Using the curated dataset, a linear model was fit and all possible log-fold change in PC value comparisons between the four condensate systems were calculated using limma functions lmFit, makeContrasts, and contrasts.fit. Finally, empirical Bayes moderation of test statistics was performed using limma function eBayes, an approach that improves inference for small sample sizes by considering estimates of variances both for each compound separately and pooled across the complete dataset[28]. We allowed an intensity-variance trend (trend=TRUE) and used robust estimation (robust=TRUE) to reduce the influence of outlier PC values. Correction for multiple comparisons was performed using the Benjamini-Hochberg

procedure. We consider a compound differentially enriched in a condensate if the fold change in PC value is greater than 10 in either direction and if the two-sided p-value is less than 0.01. Volcano plots (Extended Figure 5) are generated using these cutoffs. Fold changes for each condensate, test statistics, p-values, differentially enriched compounds, and compounds that are consistently differentially enriched in a given condensate when compared to all other condensates are reported in Supplemental Table S12 and S13. Code for this analysis is available at https://git.biohpc.swmed.edu/rosen-lab/small-molecule-properties-define-partitioning-into-biomolecular-condensates.

## Data Availability

The database used in this study is CHEMBL Database Release 32, 2023. All data are available in the main text or provided supplementary materials (Tables in MS Excel file format). All raw data is available in Dryad webserver (doi:10.5061/dryad.fxpnvx10r). All code described in the manuscript is either provided in Supplementary Materials or available on the Sigman Lab GitHub. Code for subsample correlation and differential enrichment analyses are available on the Rosen Lab Gitlab.

## Code Availability

All code described in the manuscript is either provided in Supplementary Materials or available on the Sigman Lab GitHub: https://github.com/SigmanGroup/small-molecule-partitioning. Code for subsample correlation and differential enrichment analyses are available on the Rosen Lab Gitlab: https://git.biohpc.swmed.edu/rosen-lab/small-molecule-properties-define-partitioning-into-biomolecular-condensates

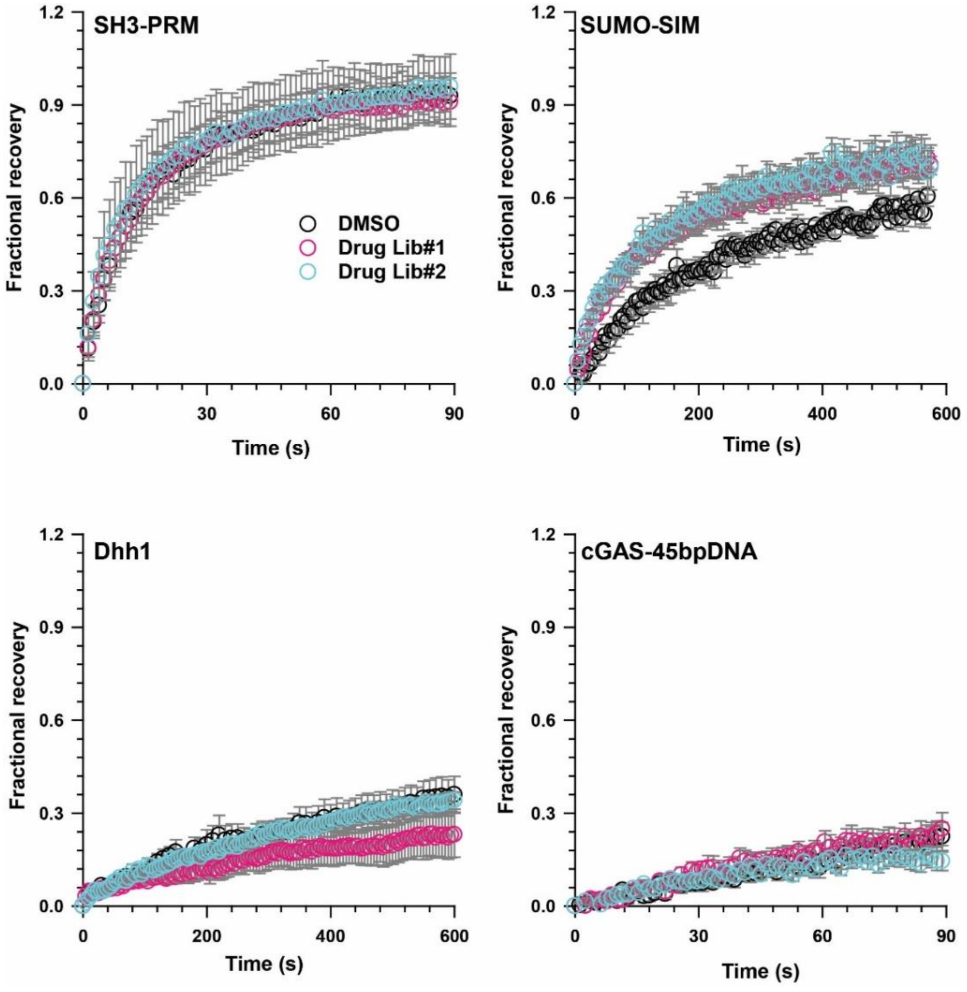


**Supplementary Figure 1:** Fluorescence Recovery After Photobleaching (FRAP) of all four condensates in the absence (DMSO) and presence of two drug sub libraries (Dark-Library #1 and Dark-Library #2, derived from standard sub libraries #1 and #2 by removing fluorescent compounds). The time of recovery and the percentage of recovery for each condensate system under different conditions are presented in a table (Extended figure 2I). Data are presented as mean values +/- SD and values are calculated from three independent sample measurements. The data show that the SH3PRM condensate was not affected by either Dark-Library, while the SUMOSIM and Dhh1 condensates both recovered slightly more slowly in the presence of one Dark-Library (a different one in each case). cGASDNA also does not appear to be affected, but the condensates recover too slowly to make a definitive comparison, having hardened during the course of the analysis. Thus, the compound libraries do not appear to substantially alter the properties of the condensates.

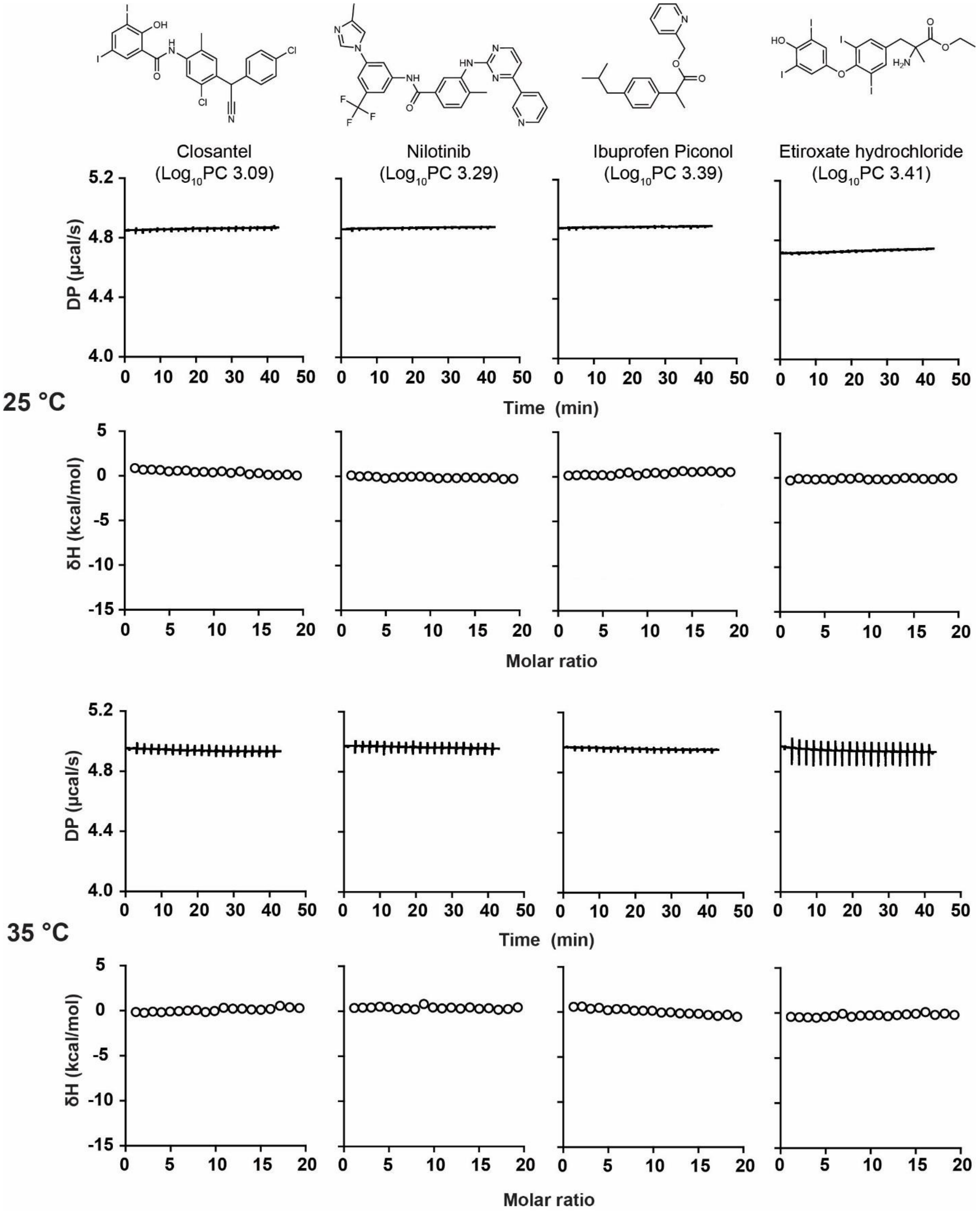
Closantel
(Log10PC 3.09)
Nilotinib
(Log10PC 3.29)
Ibuprofen Piconol
(Log10PC 3.39)
Etiroxate hydrochloride
(Log10PC 3.41)
25 °C
DP (μcal/s)
Time (min)
δH (kcal/mol)
Molar ratio
35 °C
DP (μcal/s)
Time (min)
δH (kcal/mol)
Molar ratio

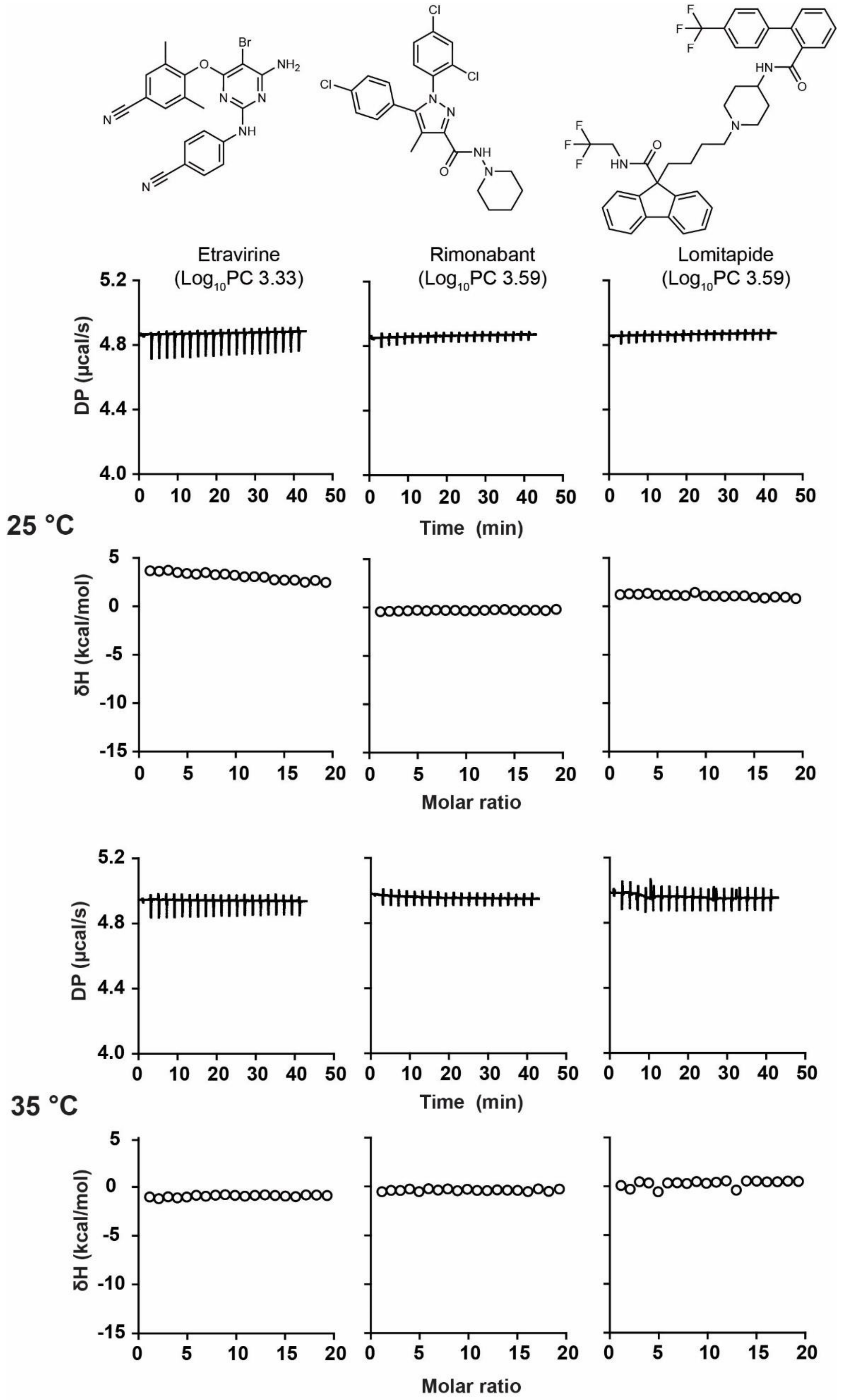
Etravirine
($Log_{10}$PC 3.33)
Rimonabant
($Log_{10}$PC 3.59)
Lomitapide
($Log_{10}$PC 3.59)
DP (µcal/s)
Time (min)
25 °C
δH (kcal/mol)
Molar ratio
DP (µcal/s)
Time (min)
35 °C
δH (kcal/mol)
Molar ratio

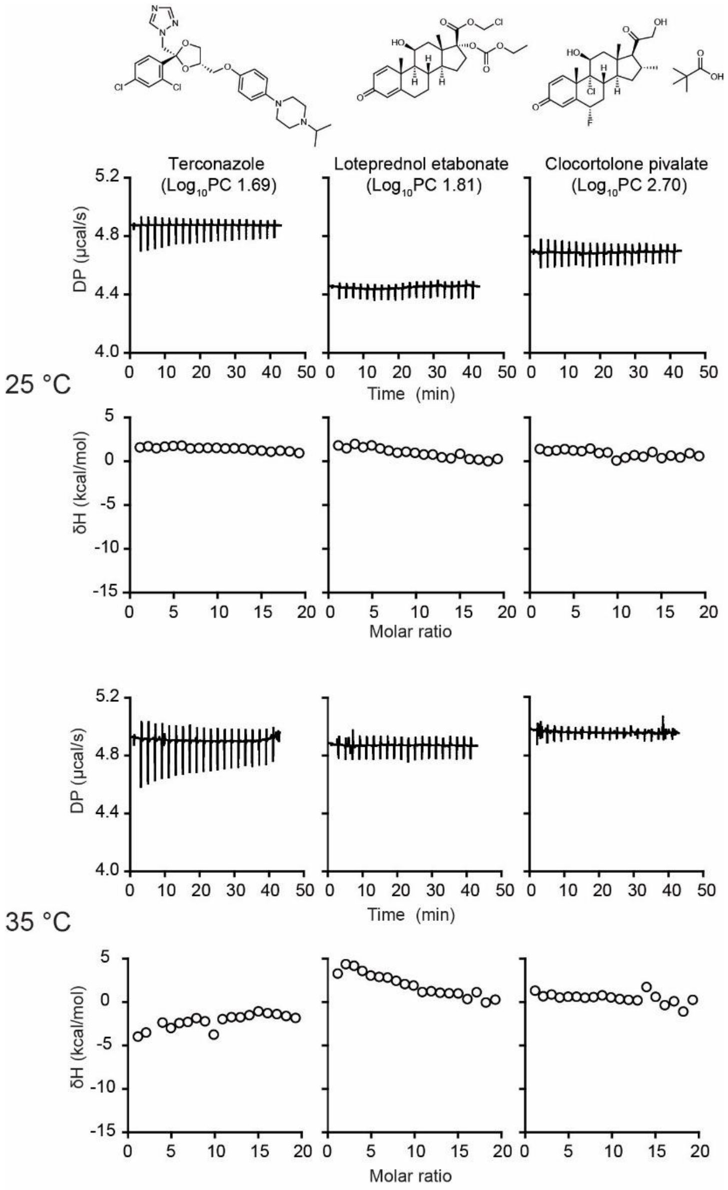
Terconazole
(Log10PC 1.69)
Loteprednol etabonate
(Log10PC 1.81)
Clocortolone pivalate
(Log10PC 2.70)
DP (µcal/s)
Time (min)
25 °C
δH (kcal/mol)
Molar ratio
35 °C

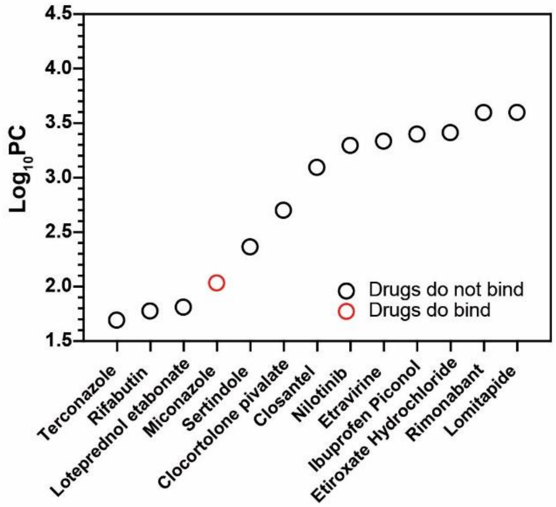
Log10PC
Drugs do not bind
Drugs do bind
Terconazole
Rifabutin
Loteprednol etabonate
Miconazole
Sertindole
Clocortolone pivalate
Closantel
Nilotinib
Etravirine
Ibuprofen Piconol
Etiroxate Hydrochloride
Rimonabant
Lomitapide

**Supplementary Figure 2:** Additional compounds do not bind SUMOSIM scaffolds under non-phase separating conditions. Raw thermograms (top row) and integrated enthalpies (bottom row) of titrations of Closantel, Nilotinib, Ibuprofen piconol, Etiroxate hydrochloride, Etravirine, Rimonabant, Lomitapide, Terconazole, Loteprednol etabonate, and Clocortolone pivalate, into 20 μM module concentrations of polySUMO + polySIM (below phase separation threshold) monitored by ITC. Titrations at 25 °C (Top panels) and 35 °C (bottom panels) for each compound. Summary of ITC data plotted against logPC for the 13 compounds examined. Only miconazole shows saturable binding (colored red).

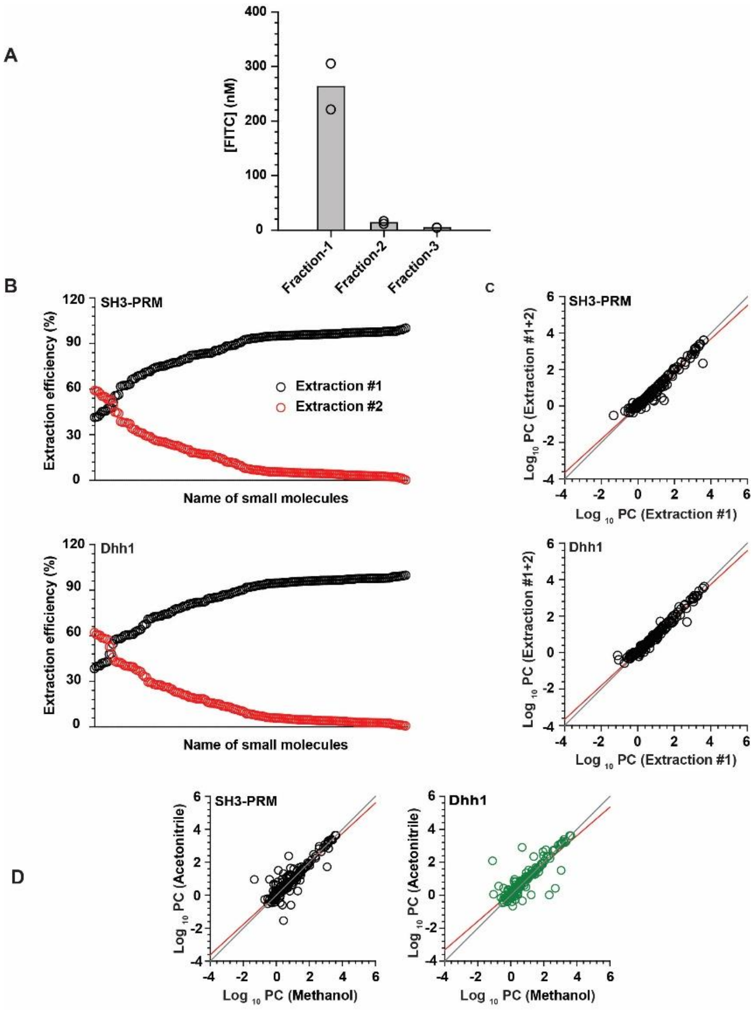


**Supplementary Figure 3: Evaluating efficiency of extraction of small molecules from protein samples.** See Materials and methods for detailed protocols describing extraction of small molecules from protein samples. A. Efficiency of three successive rounds of extraction of FITC from SH3PRM condensates. B. Efficiency of two successive rounds of extraction (assuming total from the two rounds is 100%) of small molecules in a 300-compound sub-library from SH3PRM (top) and Dhh1 (bottom) condensates using -80 °C methanol. We used the same procedure to extract small molecules from the bulk samples (not shown) to determine PC. C. Correlation between PC values calculated from two rounds of small molecule extraction versus only the first round of extraction, for SH3PRM (top) and Dhh1 (bottom) condensates. D. Correlations comparing the PC of small molecules extracted from SH3PRM (left) and Dhh1 (right) condensates using -80 °C acetonitrile *versus* -80 °C methanol.

**Table S1: List of constructs and DNA molecules used in this study.**

| No: | Name |
|---|---|
| 1 | polySUMO (SUMO-10R)<br><br>MGGSWGGSMSEEKPKEGVKTENDHINLKVAGQDGSVVQFKIKRHTPLSKLMKAYSERQGLSMRQIRFRFD<br>GQPINETDTPAQLEMEDEDTIDVFQQQTVVGGSGGSGGSGGSMSEEKPKEGVKTENDHINLKVAGQDGSV<br>VQFKIKRHTPLSKLMKAYSERQGLSMRQIRFRFDGQPINETDTPAQLEMEDEDTIDVFQQQTVVGGSGGS<br>GGSGGSMSEEKPKEGVKTENDHINLKVAGQDGSVVQFKIKRHTPLSKLMKAYSERQGLSMRQIRFRFDGQ<br>PINETDTPAQLEMEDEDTIDVFQQQTVVGGSGGSGGSGGSMSEEKPKEGVKTENDHINLKVAGQDGSVVQ<br>FKIKRHTPLSKLMKAYSERQGLSMRQIRFRFDGQPINETDTPAQLEMEDEDTIDVFQQQTVVGGSGGSGG<br>SGGSMSEEKPKEGVKTENDHINLKVAGQDGSVVQFKIKRHTPLSKLMKAYSERQGLSMRQIRFRFDGQPI<br>NETDTPAQLEMEDEDTIDVFQQQTVVGGSGGSGGSWGGSMSEEKPKEGVKTENDHINLKVAGQDGSVVQF<br>KIKRHTPLSKLMKAYSERQGLSMRQIRFRFDGQPINETDTPAQLEMEDEDTIDVFQQQTVVGGSGGSGGS<br>GGSMSEEKPKEGVKTENDHINLKVAGQDGSVVQFKIKRHTPLSKLMKAYSERQGLSMRQIRFRFDGQPIN<br>ETDTPAQLEMEDEDTIDVFQQQTVVGGSGGSGGSGGSMSEEKPKEGVKTENDHINLKVAGQDGSVVQFKI<br>KRHTPLSKLMKAYSERQGLSMRQIRFRFDGQPINETDTPAQLEMEDEDTIDVFQQQTVVGGSGGSGGSGG<br>SMSEEKPKEGVKTENDHINLKVAGQDGSVVQFKIKRHTPLSKLMKAYSERQGLSMRQIRFRFDGQPINET<br>DTPAQLEMEDEDTIDVFQQQTVVGGSGGSGGSGGSMSEEKPKEGVKTENDHINLKVAGQDGSVVQFKIKR<br>HTPLSKLMKAYSERQGLSMRQIRFRFDGQPINETDTPAQLEMEDEDTIDVFQQQTVVGGSGGSENLYFQ |

| | |
|---|---|
| **2** | **polySIM (SIM-10R)**<br><br>MGGSWGGSKVDVIDLTIESSSDEEEDPPAKRGGSGGSGGSGGSKVDVIDLTIESSSDEEEDPPAKRGGSG<br>GSGGSGGSKVDVIDLTIESSSDEEEDPPAKRGGSGGSGGSGGSKVDVIDLTIESSSDEEEDPPAKRGGSG<br>GSGGSGGSKVDVIDLTIESSSDEEEDPPAKRGGSGGSGGSGGSKVDVIDLTIESSSDEEEDPPAKRGSKV<br>DVIDLTIESSSDEEEDPPAKRGGSGGSGGSGGSKVDVIDLTIESSSDEEEDPPAKRGGSGGSGGSGGSKV<br>DVIDLTIESSSDEEEDPPAKRGGSGGSGGSGGSKVDVIDLTIESSSDEEEDPPAKR |
| **3** | **PolySH3 (SH3-5R)**<br><br>MDLNMPAYVKFNYMAEREDELSLIKGTKVIVMEKSSDGWWRGSYNGQVGWFPSNYVTEEGDSPLASGAGG<br>SEGGGSEGGTSGATDLNMPAYVKFNYMAEREDELSLIKGTKVIVMEKSSDGWWRGSYNGQVGWFPSNYVT<br>EEGDSPLASGAGGSEGGGSEGGTSGATHMDLNMPAYVKFNYMAEREDELSLIKGTKVIVMEKSSDGWWRG<br>SYNGQVGWFPSNYVTEEGDSPLASGAGGSEGGGSEGGTSGATDLNMPAYVKFNYMAEREDELSLIKGTKV<br>IVMEKSSDGWWRGSYNGQVGWFPSNYVTEEGDSPLASGAGGSEGGGSEGGTSGATDLNMPAYVKFNYMAE<br>REDELSLIKGTKVIVMEKSSDGWWRGSYNGQVGWFPSNYVTEEGDSPLGGGSENLYFQ |
| **4** | **polyPRM (PRM-5R)**<br><br>KGGSWGGSKKKKTAPTPPKRSGGSGGSGGSGGSKKKKTAPTPPKRSGGSGGSGGSGGSKKKKTAPTPPKR<br>SGGSGGSGGSGGSKKKKTAPTPPKRSGGSGGSGGSGGSKKKKTAPTPPKRSGGSGSENLYFQ |

| | |
|---|---|
| 5 | **Dhh1**<br><br>MGSINNNFNTNNNSNTDLDRDWKTALNIPKKDTRPQTDDVLNTKGNTFEDFYLKRELLMGIFEAGFEKPS<br>PIQEEAIPVAITGRDILARAKNGTGKTAAFVIPTLEKVKPKLNKIQALIMVPTRELALQTSQVVRTLGKH<br>CGISCMVTTGGTNLRDDILRLNETVHILVGTPGRVLDLASRKVADLSDCSLFIMDEADKMLSRDFKTIIE<br>QILSFLPPTHQSLLFSATFPLTVKEFMVKHLHKPYEINLMEELTLKGITQYYAFVEERQKLHCLNTLFSK<br>LQINQAIIFCNSTNRVELLAKKITDLGYSCYYSHARMKQQERNKVFHEFRQGKVRTLVCSDLLTRGIDIQ<br>AVNVVINFDFPKTAETYLHRIGRSGRFGHLGLAINLINWNDRFNLYKIEQELGTEIAAIPATIDKSLYVA<br>ENDETVPVPFPIEQQSYHQQAIPQQQLPSQQQFAIPPQQHHPQFMVPPSHQQQQAYPPPQMPSQQGYPPQ<br>QEHFMAMPPGQSQPQY |
| 6 | **Human cGAS**<br><br>MQPWHGKAMQRASEAGATAPKASARNARGAPMDPTESPAAPEAALPKAGKFGPARKSGSRQKKSAPDTQE<br>RPPVRATGARAKKAPQRAQDTQPSDATSAPGAEGLEPPAAREPALSRAGSCRQRGARCSTKPRPPPGPWD<br>VPSPGLPVSAPILVRRDAAPGASKLRAVLEKLKLSRDDISTAAGMVKGVVDHLLLRLKCDSAFRGVGLLN<br>TGSYYEHVKISAPNEFDVMFKLEVPRIQLEEYSNTRAYYFVKFKRNPKENPLSQFLEGEILSASKMLSKF<br>RKIIKEEINDIKDTDVIMKRKRGGSPAVTLLISEKISVDITLALESKSSWPASTQEGLRIQNWLSAKVRK<br>QLRLKPFYLVPKHAKEGNGFQEETWRLSFSHIEKEILNNHGKSKTCCENKEEKCCRKDCLKLMKYLLEQL<br>KERFKDKKHLDKFSSYHVKTAFFHVCTQNPQDSQWDRKDLGLCFDNCVTYFLQCLRTEKLENYFIPEFNL<br>FSSNLIDKRSKEFLTKQIEYERNNEFPVFDEF |
| 7 | **45bp Immunostimulatory DNA (ISD) Forward**<br><br>5′-TACAGATCTACTAGTGATCTATGACTGATCTGTACATGATCTACA-3′ |

| | |
|---|---|
| | **45bp Immunostimulatory DNA (ISD) Reverse**<br><br>**5′-TGTAGATCATGTACAGATCAGTCATAGATCACTAGTAGATCTGTA-3′** |

## Method-only References


1 Banani, S. F. *et al.* Compositional Control of Phase-Separated Cellular Bodies. *Cell* **166**, 651-663, doi:10.1016/j.cell.2016.06.010 (2016).

2 Li, P. *et al.* Phase transitions in the assembly of multivalent signalling proteins. *Nature* **483**, 336-340, doi:10.1038/nature10879 (2012).

3 Du, M. & Chen, Z. J. DNA-induced liquid phase condensation of cGAS activates innate immune signaling. *Science* **361**, 704-709, doi:10.1126/science.aat1022 (2018).

4 Currie, S. L. *et al.* Quantitative reconstitution of yeast RNA processing bodies. *bioRxiv*, 2022.2008.2013.503854, doi:10.1101/2022.08.13.503854 (2022).

5 Peeples, W. & Rosen, M. K. Mechanistic dissection of increased enzymatic rate in a phase-separated compartment. *Nat Chem Biol* **17**, 693-702, doi:10.1038/s41589-021-00801-x (2021).

6 Sellick, C. A., Hansen, R., Stephens, G. M., Goodacre, R. & Dickson, A. J. Metabolite extraction from suspension-cultured mammalian cells for global metabolite profiling. *Nat Protoc* **6**, 1241-1249, doi:10.1038/nprot.2011.366 (2011).

7 Currie, S. L. *et al.* Quantitative reconstitution of yeast RNA processing bodies. *Proc Natl Acad Sci U S A* **120**, e2214064120, doi:10.1073/pnas.2214064120 (2023).

8 Freibaum, B. D., Messing, J., Yang, P., Kim, H. J. & Taylor, J. P. High-fidelity reconstitution of stress granules and nucleoli in mammalian cellular lysate. *J Cell Biol* **220**, doi:10.1083/jcb.202009079 (2021).

9 Good, M. C. & Heald, R. Preparation of Cellular Extracts from Xenopus Eggs and Embryos. *Cold Spring Harb Protoc* **2018**, doi:10.1101/pdb.prot097055 (2018).

10 Yuan, M., Breitkopf, S. B., Yang, X. & Asara, J. M. A positive/negative ion-switching, targeted mass spectrometry-based metabolomics platform for bodily fluids, cells, and fresh and fixed tissue. *Nat Protoc* **7**, 872-881, doi:10.1038/nprot.2012.024 (2012).

11 Tautenhahn, R. *et al.* An accelerated workflow for untargeted metabolomics using the METLIN database. *Nature biotechnology* **30**, 826-828 (2012).

12 RCoreTeam. R: A Language and Environment for Statistical Computing. doi:https://www.R-project.org (2023).

13 LigPrep. Schrödinger Release 2022-3: LigPrep, Schrödinger, LLC, New York, NY, 2021. (2021).

14 Lu, C. *et al.* OPLS4: Improving Force Field Accuracy on Challenging Regimes of Chemical Space. *Journal of Chemical Theory and Computation* **17**, 4291-4300, doi:10.1021/acs.jctc.1c00302 (2021).

15 Epik. Schrödinger Release 2022-3: Protein Preparation Wizard; Schrödinger, LLC, New York, NY, 2021; Impact, Schrödinger, LLC, New York, NY; Prime, Schrödinger, LLC, New York, NY, 2021. (2021).

16 QikProp. Schrödinger Release 2022-4: QikProp, Schrödinger, LLC, New York, NY, 2021. (2021).

17 McInnes, L., Healy, J. & Melville, J. Umap: Uniform manifold approximation and projection for dimension reduction. *arXiv preprint arXiv:1802.03426* (2018).

18 Campello, R. J., Moulavi, D. & Sander, J. in *Pacific-Asia conference on knowledge discovery and data mining.* 160-172 (Springer).

19 Hersey, A. *CHEMBL Database Release 32*, 2023).

20 Huang, K. *et al.* Therapeutics data commons: Machine learning datasets and tasks for drug discovery and development. *arXiv preprint arXiv:2102.09548* (2021).

21 Bento, A. P. *et al.* An open source chemical structure curation pipeline using RDKit. *Journal of Cheminformatics* **12**, 1-16 (2020).

22 Seabold, S. & Perktold, J. in *Proc 9th Python Sci Conf.* 61.

23 Morgan, H. L. The Generation of a Unique Machine Description for Chemical Structures-A Technique Developed at Chemical Abstracts Service. *Journal of Chemical Documentation* **5**, 107-113, doi:10.1021/c160017a018 (1965).

24 Rogers, D. & Hahn, M. Extended-Connectivity Fingerprints. *Journal of Chemical Information and Modeling* **50**, 742-754, doi:10.1021/ci100050t (2010).

25 Riniker, S. & Landrum, G. A. Open-source platform to benchmark fingerprints for ligand-based virtual screening. *Journal of cheminformatics* **5**, 26 (2013).

26 Moriwaki, H., Tian, Y.-S., Kawashita, N. & Takagi, T. Mordred: a molecular descriptor calculator. *Journal of cheminformatics* **10**, 1-14 (2018).

27 Messner, C. B. *et al.* The proteomic landscape of genome-wide genetic perturbations. *Cell* **186**, 2018-2034.e2021, doi:https://doi.org/10.1016/j.cell.2023.03.026 (2023).

28 Ritchie, M. E. *et al.* limma powers differential expression analyses for RNA-sequencing and microarray studies. *Nucleic Acids Research* **43**, e47-e47, doi:10.1093/nar/gkv007 (2015).
