# Supplementary material for: Small-molecule properties define partitioning into biomolecular condensates
Source: Nat Chem. 2024 Sep 13;16(11):1794–802. doi: 10.1038/s41557-024-01630-w (PMC11527791; doi:10.1038/s41557-024-01630-w)
Supplement: Supplementary file 2 — Reporting Summary [file 41557_2024_1630_MOESM2_ESM.pdf]

nature portfolio

Corresponding author(s): Michael K. Rosen
Matthew S. Sigman

Last updated by author(s): Jul 18, 2024

Reporting Summary

Nature Portfolio wishes to improve the reproducibility of the work that we publish. This form provides structure for consistency and transparency in reporting. For further information on Nature Portfolio policies, see our Editorial Policies and the Editorial Policy Checklist.

Statistics

For all statistical analyses, confirm that the following items are present in the figure legend, table legend, main text, or Methods section.

n/a	Confirmed	
☐	☒	The exact sample size (*n*) for each experimental group/condition, given as a discrete number and unit of measurement
☐	☒	A statement on whether measurements were taken from distinct samples or whether the same sample was measured repeatedly
☐	☒	The statistical test(s) used AND whether they are one- or two-sided<br>*Only common tests should be described solely by name; describe more complex techniques in the Methods section.*
☒	☐	A description of all covariates tested
☒	☐	A description of any assumptions or corrections, such as tests of normality and adjustment for multiple comparisons
☐	☒	A full description of the statistical parameters including central tendency (e.g. means) or other basic estimates (e.g. regression coefficient) AND variation (e.g. standard deviation) or associated estimates of uncertainty (e.g. confidence intervals)
☐	☒	For null hypothesis testing, the test statistic (e.g. *F*, *t*, *r*) with confidence intervals, effect sizes, degrees of freedom and *P* value noted<br>*Give P values as exact values whenever suitable.*
☒	☐	For Bayesian analysis, information on the choice of priors and Markov chain Monte Carlo settings
☒	☐	For hierarchical and complex designs, identification of the appropriate level for tests and full reporting of outcomes
☐	☒	Estimates of effect sizes (e.g. Cohen's *d*, Pearson's *r*), indicating how they were calculated

Our web collection on statistics for biologists contains articles on many of the points above.

Software and code

Policy information about availability of computer code

Data collection	Mass Spec data collection:Analyst 1.7.2 software (AB Sciex)<br>ITC Data collection: MicroCal PEAQ-ITC Control v1.4<br>Microscopy data collection: Leica Application Suite X (LAS X) version 3.5.7.2325
Data analysis	MS Data analysis: SCIEX OS 2.1 (AB Sciex),<br>Graph and data analysis:SigmaPlot 10.0 for Windows 10 , MS Excel v2202<br>ITC Data analysis: MicroCal PEAQ-ITC Analysis v1.4<br>Image analysis: Fiji (Image J2) version 1.53C<br>R version 4.2.3 R Core Team 2023<br>Statsmodels python package 0.14.1<br>Limma Version 3.54.2<br>Github: https://github.com/SigmanGroup/small-molecule-partitioning.<br>Gitlab: https://git.biohpc.swmed.edu/rosen-lab/small-molecule-properties-define-partitioning-into-biomolecular-condensates.

For manuscripts utilizing custom algorithms or software that are central to the research but not yet described in published literature, software must be made available to editors and reviewers. We strongly encourage code deposition in a community repository (e.g. GitHub). See the Nature Portfolio guidelines for submitting code & software for further information.

Data

Policy information about availability of data

All manuscripts must include a data availability statement. This statement should provide the following information, where applicable:

- Accession codes, unique identifiers, or web links for publicly available datasets
- A description of any restrictions on data availability
- For clinical datasets or third party data, please ensure that the statement adheres to our policy

The database used in this study is CHEMBL Database Release 32, 2023. All data are available in the main text or provided supplementary materials (Tables in MS Excel file format). All raw data is available in Dryad webserver (doi:10.5061/dryad.fxpnvx10r). All code described in the manuscript is either provided in Supplementary Materials or available on the Sigman Lab Github. Code for subsample correlation and differential enrichment analyses are available on the Rosen Lab Gitlab:

Human research participants

Policy information about studies involving human research participants and Sex and Gender in Research.

Reporting on sex and gender	N/A
Population characteristics	N/A
Recruitment	N/A
Ethics oversight	N/A

Note that full information on the approval of the study protocol must also be provided in the manuscript.

Field-specific reporting

Please select the one below that is the best fit for your research. If you are not sure, read the appropriate sections before making your selection.

☒ Life sciences ☐ Behavioural & social sciences ☐ Ecological, evolutionary & environmental sciences

For a reference copy of the document with all sections, see nature.com/documents/nr-reporting-summary-flat.pdf

Life sciences study design

All studies must disclose on these points even when the disclosure is negative.

Sample size	No sample size calculations were performed. Sample sizes were chosen as large as possible and adequate statistics are reported through out the manuscript.
Data exclusions	No experimental data were excluded
Replication	Reported experiments were repeated in at least 3 replicates with consistent results. The error bars in all experiments represent the standard error calculated from all the replicates.
Randomization	All samples were analyzed and reported, individually there was no allocation into any groups
Blinding	Blinding was not necessary because there are no subjective assessments or treatments.

Reporting for specific materials, systems and methods

We require information from authors about some types of materials, experimental systems and methods used in many studies. Here, indicate whether each material, system or method listed is relevant to your study. If you are not sure if a list item applies to your research, read the appropriate section before selecting a response.

Materials & experimental systems

n/a	Involved in the study
☒	☐ Antibodies
☐	☒ Eukaryotic cell lines
☒	☐ Palaeontology and archaeology
☒	☐ Animals and other organisms
☒	☐ Clinical data
☒	☐ Dual use research of concern

Methods

n/a	Involved in the study
☒	☐ ChIP-seq
☒	☐ Flow cytometry
☒	☐ MRI-based neuroimaging

Eukaryotic cell lines

Policy information about cell lines and Sex and Gender in Research

Cell line source(s)	U2OS cell lines, The original commercial source of U2OS cells is the American Type Culture Collection (ATCC)
Authentication	N.D
Mycoplasma contamination	Routine mycoplasma tested showed all cell lines were free of mycoplasma contamination.
Commonly misidentified lines (See ICLAC register)	U2OS Cell lines are not in the list of commonly misidentified cell lines
