# Supplementary material for: Small-molecule properties define partitioning into biomolecular condensates
Source: Nat Chem. 2024 Sep 13;16(11):1794–802. doi: 10.1038/s41557-024-01630-w (PMC11527791; doi:10.1038/s41557-024-01630-w)

Uncropped gel pictures (Associated with Extended Figure 2A, 2C, 2E, and 2G)

cGAS-DNA_Droplet_Fraction

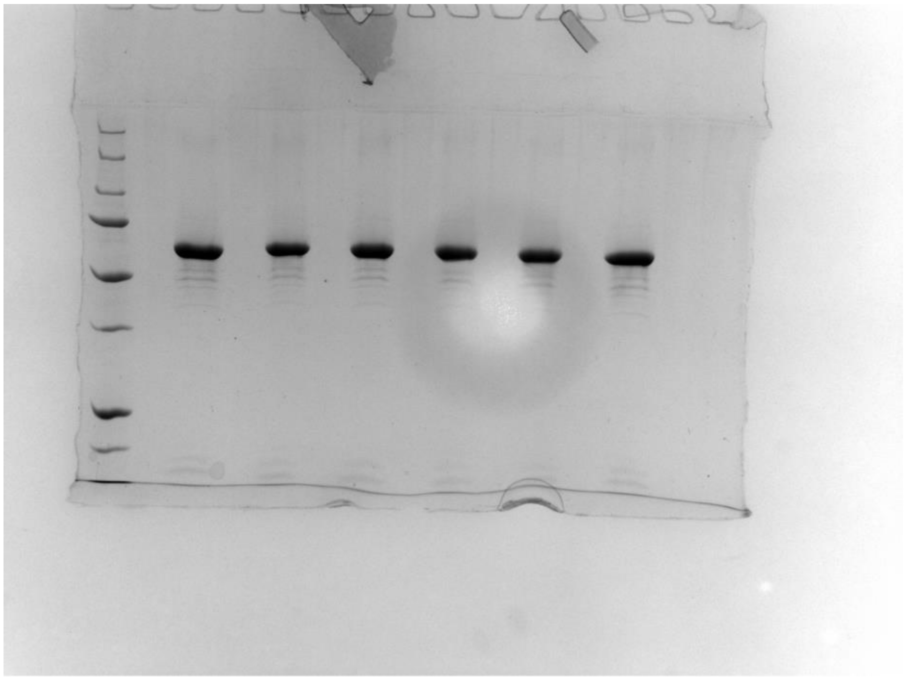

cGAS-DNA_Solution_Fraction

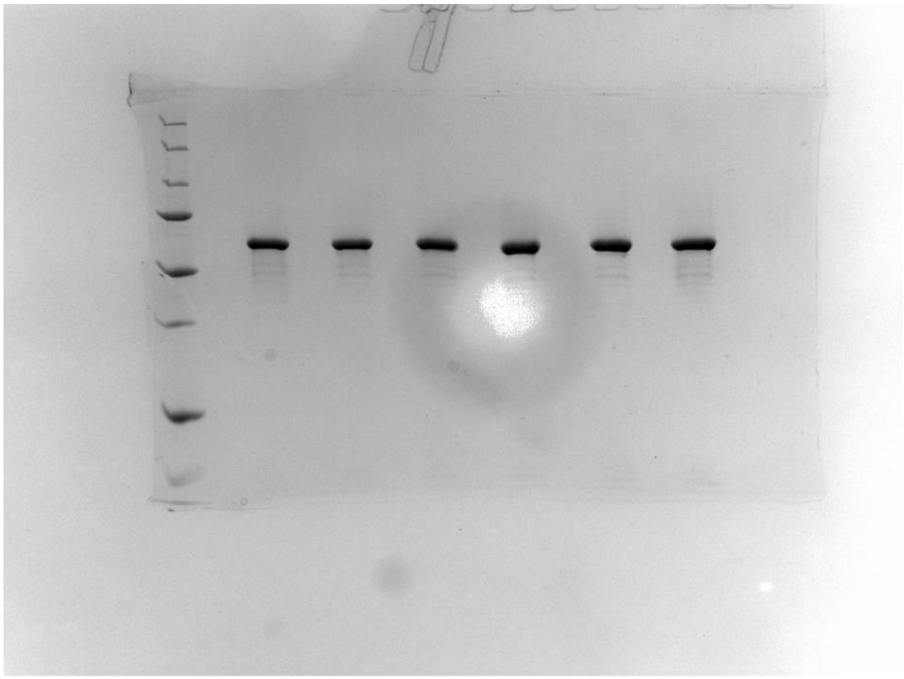

SUMOSIM_Droplet_Fraction

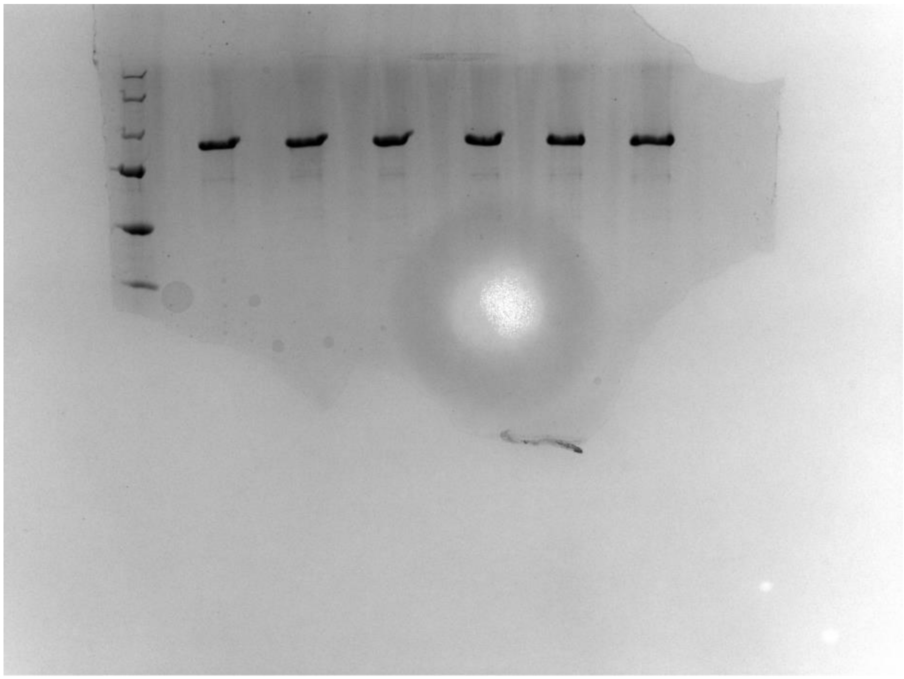

SUMOSIM_Solution_Fraction

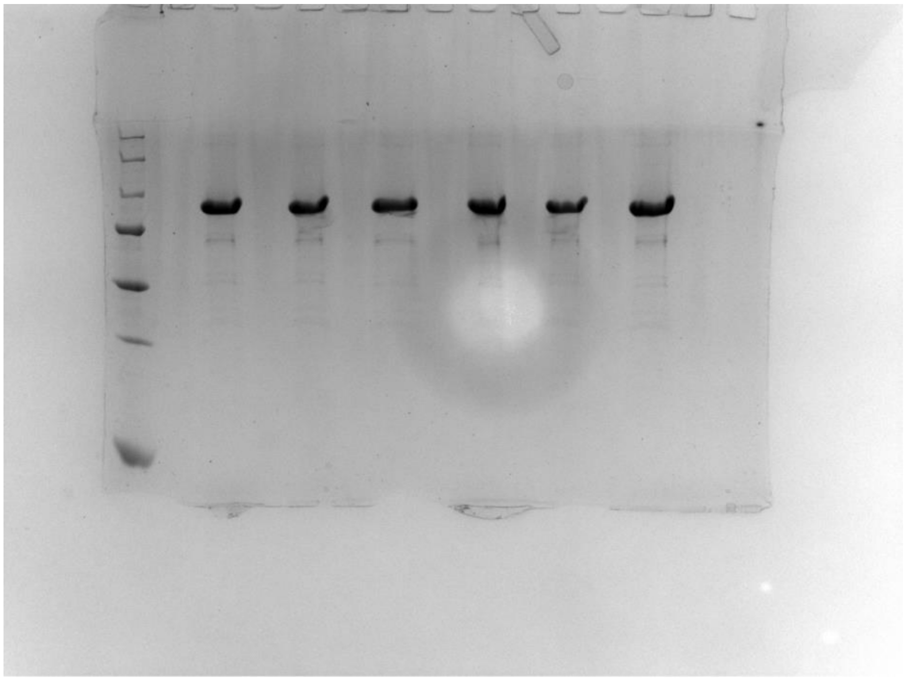

SH3PRM_Droplet_Fraction

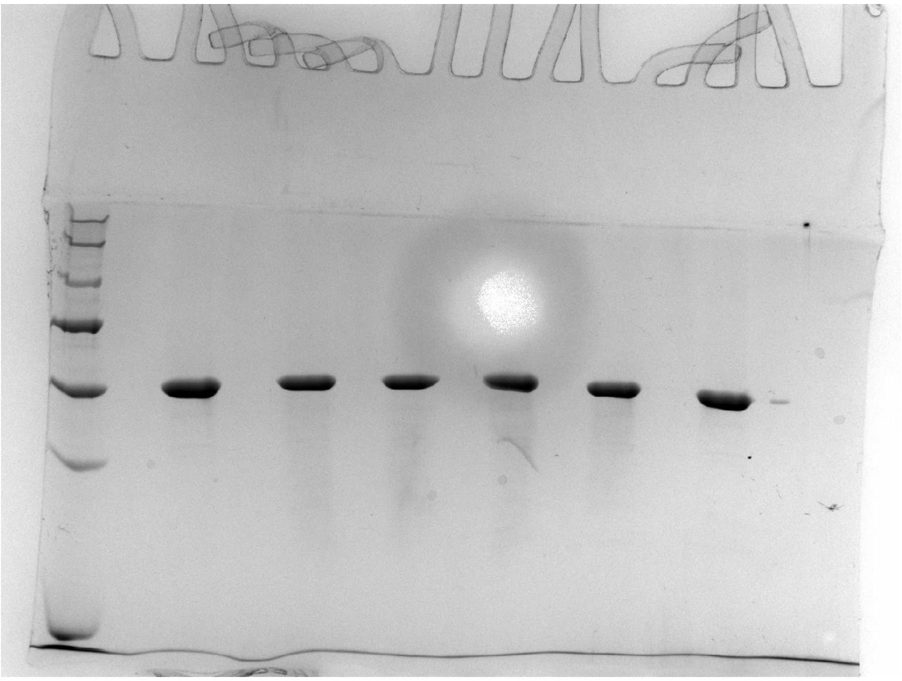

SH3PRM_Solution_Fraction

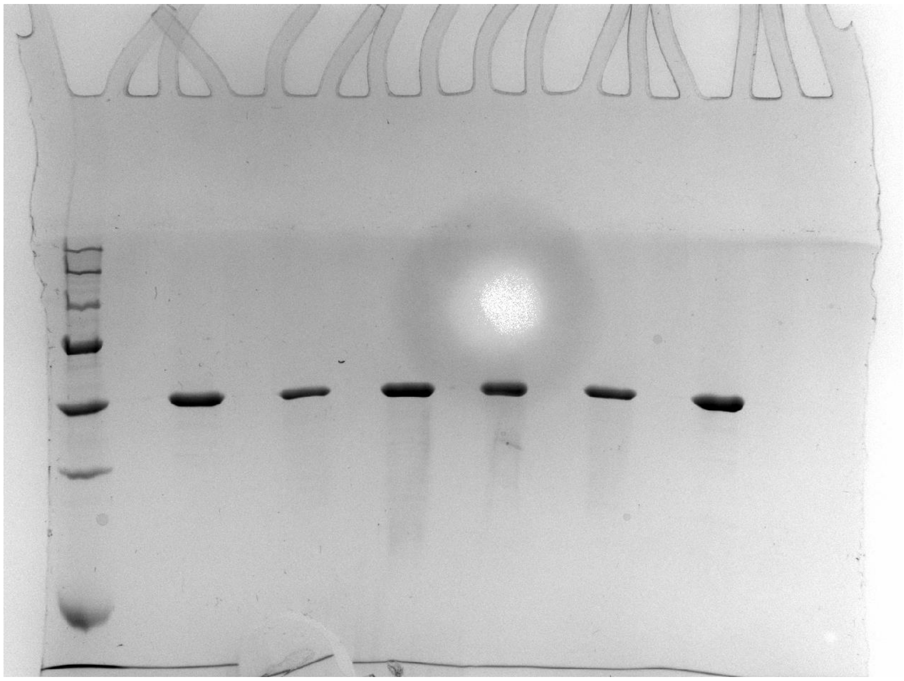

Dhh1_Droplet_Fraction

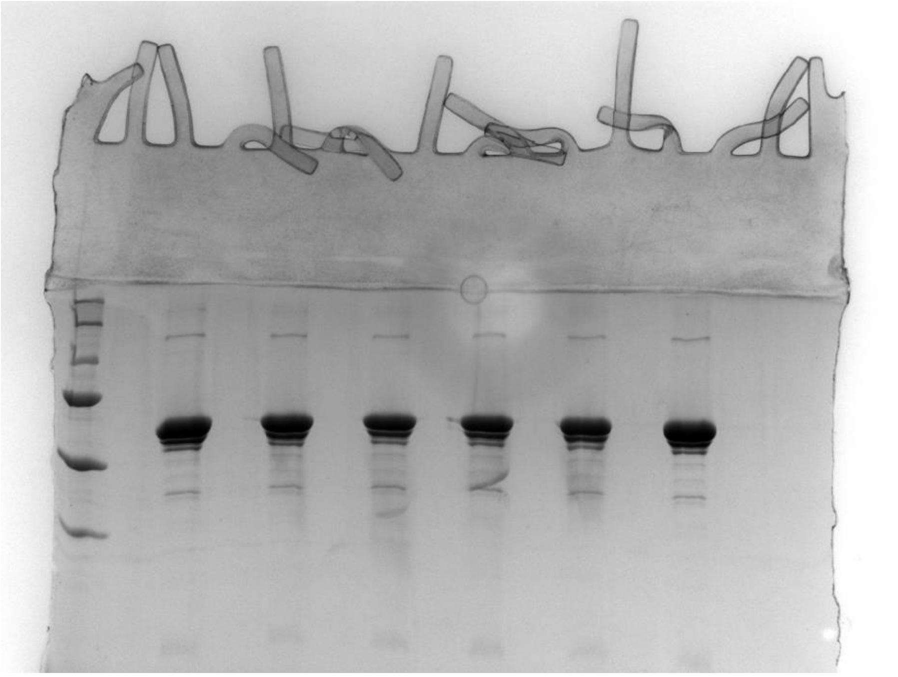

Dhh1_Solution_Fraction

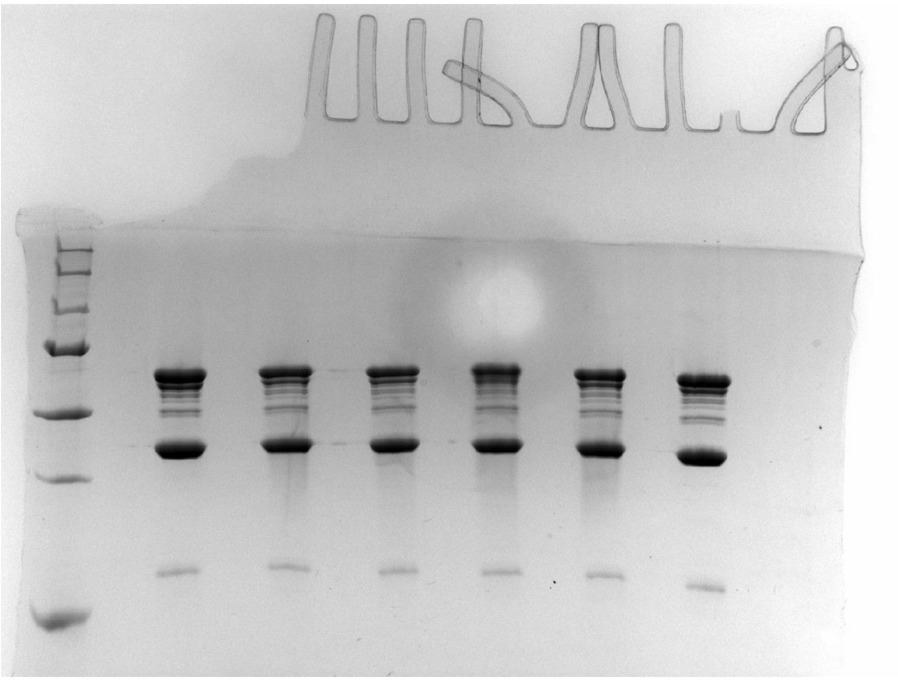

Supplement: Supplementary file 22 — Excel files used for each plot. [file 41557_2024_1630_MOESM22_ESM.zip › Extended_Figure_2/Extended Data Figure_2A-C-E_G_SourceData.pdf]
